# Multi-trait GWAS using imputed high-density genotypes from whole-genome sequencing identifies genes associated with body traits in Nile tilapia

**DOI:** 10.1186/s12864-020-07341-z

**Published:** 2021-01-15

**Authors:** Grazyella M. Yoshida, José M. Yáñez

**Affiliations:** 1 Facultad de Ciencias Veterinarias y Pecuarias, Universidad de Chile, Santiago, Chile; 2 Núcleo Milenio INVASAL, Concepción, Chile

**Keywords:** Body traits, Genome-wide association study, Genotype imputation, Quantitative trait loci, *Oreochromis niloticus*, Multi-trait

## Abstract

**Background:**

Body traits are generally controlled by several genes in vertebrates (i.e. polygenes), which in turn make them difficult to identify through association mapping. Increasing the power of association studies by combining approaches such as genotype imputation and multi-trait analysis improves the ability to detect quantitative trait loci associated with polygenic traits, such as body traits.

**Results:**

A multi-trait genome-wide association study (mtGWAS) was performed to identify quantitative trait loci (QTL) and genes associated with body traits in Nile tilapia (*Oreochromis niloticus*) using genotypes imputed to whole-genome sequences (WGS). To increase the statistical power of mtGWAS for the detection of genetic associations, summary statistics from single-trait genome-wide association studies (stGWAS) for eight different body traits recorded in 1309 animals were used. The mtGWAS increased the statistical power from the original sample size from 13 to 44%, depending on the trait analyzed. The better resolution of the WGS data, combined with the increased power of the mtGWAS approach, allowed the detection of significant markers which were not previously found in the stGWAS. Some of the lead single nucleotide polymorphisms (SNPs) were found within important functional candidate genes previously associated with growth-related traits in other terrestrial species. For instance, we identified SNP within the *α1,6-fucosyltransferase* (*FUT8*), *solute carrier family 4 member 2* (*SLC4A2*), *A disintegrin and metalloproteinase with thrombospondin motifs 9* (*ADAMTS9*) and *heart development protein with EGF like domains 1* (*HEG1*) genes, which have been associated with average daily gain in sheep, osteopetrosis in cattle, chest size in goats, and growth and meat quality in sheep, respectively.

**Conclusions:**

The high-resolution mtGWAS presented here allowed the identification of significant SNPs, linked to strong functional candidate genes, associated with body traits in Nile tilapia. These results provide further insights about the genetic variants and genes underlying body trait variation in cichlid fish with high accuracy and strong statistical support.

**Supplementary Information:**

The online version contains supplementary material available at 10.1186/s12864-020-07341-z.

## Background

Tilapia is one of the most important fish species cultivated in the world, and is currently farmed in more than 125 countries. Total farmed finfish production reached 54.3 million tons globally in 2018, and Nile tilapia (*Oreochromis niloticus*) represented 8.3% of this volume [1]. Tilapia is generally sold as whole fish or fillets, making body traits, such as body and fillet weight, among the most economically important traits for this species. In fact, body size traits represent the primary breeding objective in genetic improvement programs for tilapia and other aquaculture species [2]. The most important body traits in Nile tilapia are body weight measured at a specific age (e.g. body weight at harvest), fillet weight or fillet yield (fillet weight/body weight). These traits show heritability values ranging from 0.06 to 0.48, when using pedigree-based estimates [3–9]. Previous studies have estimated high values of genetic correlations between harvest weight and fillet weight (> 0.96) and moderate to high values between harvest weight and fillet yield (0.21 to 0.74) [7, 9, 10], suggesting that is not possible to improve fillet traits independently of body weight [11]. Although, previous reports have also identified negative or null genetic correlation between harvest weight and fillet yield [12], which suggests the importance of assessing these relationships on each particular population. Other body traits which have been proposed as selection criteria to generate more profitable commercial fish populations, are reduced waste (sum of bones, viscera, head, and fins) and carcass weight, due to their higher heritability values, less correlation to body weight, compared to fillet weight, and null or even favourable impact on fillet yield [13, 14].

The availability of a chromosome-level reference genome assembly [15] and high-throughput whole-genome sequencing (WGS) methods [16, 17], have allowed for the assessment of genetic variation of different Nile tilapia populations at a genome-wide level and the recent development of single nucleotide polymorphism (SNP) panels [18, 19]. The availability of Nile tilapia SNP panels made it possible to use modern molecular breeding approaches; including mapping of quantitative trait loci (QTL) through genome-wide association studies (GWAS), marker-assisted selection (MAS) and genomic selection [20, 21]. The GWAS approach evaluates the association between genotypes and phenotypes, with both sources of information available for a large number of individuals. This method captures the linkage disequilibrium (LD) between markers and causative mutations that tend to be inherited together across generations [22]. GWAS has been applied to provide insights into both the genetic architecture and loci underpinning the genetic variation of growth-related traits in different finfish species, including Atlantic salmon and catfish [23–26], using high-density SNPs arrays (ranging from 108 K to 218 K SNPs) and, more recently, Nile tilapia by using a medium-density (50 K) SNP array [20]. These studies revealed the polygenic nature of growth-related traits and identified some genes harboring significant SNPs, which are well-known to be involved in growth and bone development, including *meprin A subunit beta-like* (*MEP1A*), *fibroblast growth factors* (*FGF*), *disintegrin and metalloproteinase domain 12* (*ADAM12*), *myosin light chain kinase* (*MYLK*) and *transforming growth-factor beta receptor type 3* (*TGFBR3*).

The use of ultra-high-density SNPs or WGS can improve the accuracy and power of GWAS to detect QTLs associated with complex traits [27–30]. Although the cost of WGS is rapidly decreasing, it is still expensive to sequence all available phenotyped individuals in a GWAS design. To solve this, genotype imputation to WGS data can be successfully implemented to detect putative causal loci in a cost-efficient manner. Previous studies using imputed genotypes from WGS for GWAS have been reported in cattle [27, 28], pigs [29, 30] and sheep [31]. In addition, new strategies such as multi-trait GWAS (mtGWAS) analysis are required to increase the power to detect QTL through GWAS [32]. mtGWAS improves the power of GWAS through the incorporation of summary information contained in the output of single-trait GWAS (stGWAS). Thus, mtGWAS jointly exploits information from genetically correlated traits to increase statistical power, due to fact that the true SNP effects and their estimated error may be correlated across traits. For instance, multi-trait approaches have been implemented in pertinent software, e.g. MTAG v0.9.0 [33], and successfully applied to boost the discovery of genetic variants associated with important traits in humans [34–36].

To the best of our knowledge, no previous studies have shown the use of imputation to high-density SNP genotypes, in a combination with mtGWAS, to uncover putative causative genetic variants associated with body traits in aquaculture species. The objective of this study was to use mtGWAS and high-density SNP genotypes to increase the accuracy and power to identify both QTLs and genes associated with eight body traits in Nile tilapia.

## Results

### Descriptive statistics, quality control and genetic parameters

A total of 1309 animals averaging 370 days-old were phenotyped and genotyped. Average, standard deviation, minimum and maximum phenotypic values for average daily gain (ADG), body weight at harvest (BWH), waste weight (WW), head weight (HW), gutted head-on weight (HON), body length at harvest (BLH), fillet weight (FW) and fillet yield (FY) are reported in Table 1. The coefficient of variation ranged between 6.86 and 27.47%, with the lowest and the highest values calculated for trait FY and FW, respectively.

**Table 1:** Descriptive statistics for phenotypic values of body traits recorded in a breeding Nile tilapia population

Traits	Mean	CV (%)	SD	Min	Max
AT (days)	113	15.32	17	76	160
Age (days)	370	5.41	20	330	427
BWT	32.97	78.95	26.03	6.00	266.00
ADG	3.54	26.27	0.93	0.69	6.29
BWH	943.98	26.01	245.54	198.00	1654.00
WW	642.84	25.76	165.60	146.00	1139.00
HW	245.62	24.11	59.23	69.00	469.00
HON	556.19	26.86	149.39	108.00	993.00
BLH	27.59	9.28	2.56	17.00	37.00
FW	300.93	27.47	82.67	85.00	528.00
FY	31.76	6.86	2.18	20.00	42.04

*AT* age at tagging, *BWT* body weight at tagging (g), *ADG* average daily gain (g), *BWH* body weight at harvest (g), *WW* waste weight (g), *HW* head weight (g), *HON* gutted head-on weight (g), *BLH* body length at harvest (cm), *FW* fillet weight (g), *FY* fillet yield (%)

For WGS, the call-rate parameter excluded the highest number of SNPs (~ 12 million), whereas MAF discarded ~ 7.8 million and ~ 253 K SNPs, for WGS and imputed WGS data, respectively. The HWE filter discarded a low number of markers, ~ 1.8 million for WGS and 79 K for imputed WGS data, respectively. After quality control applied to the 50 K SNP chip, 5905, 4114 and 3665 SNPs were removed by HWE, MAF and genotyping call-rate filters, respectively, 29,587 SNPs remained for subsequent analyses. After applying sample call-rate, all samples in both WGS and 50 K SNP chip were retained (Supplementary Table 1).

**Fig. 1:**
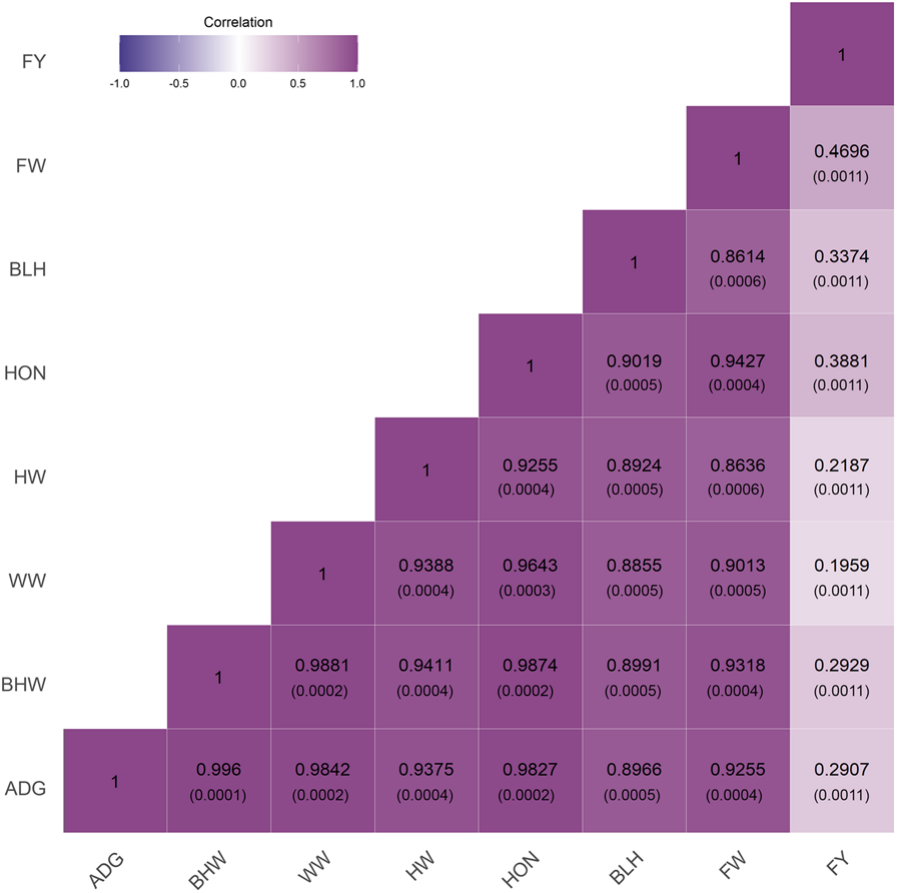
Correlation of SNP effects (standard error) among eight body traits in Nile tilapia. ADG: average daily gain (g); BWH: body weight at harvest (g); WW: waste weight (g); HW: head weight (g); HON: gutted head-on weight (g); BLH: body length at harvest (cm); FW: fillet weight (g); FY: fillet yield (%)

Heritability estimates calculated using the SNP-based genomic-relationship matrix (GRM) constructed with about 1 million markers ranged from 0.21 to 0.45 for the body traits analyzed here, with the lowest and the highest value determined for FY and HW, respectively (Table 2). The correlation of SNP effects among all body traits analyzed here ranged from 0.20 to 1.00, with small values only reported for correlations between FY and the rest of the traits (Fig. 1).

**Table 2:** Genetic parameters and comparison of association results between single- and multi-trait GWAS for Nile tilapia

Trait	${\boldsymbol{\sigma}}_{\boldsymbol{a}}^{\mathbf{2}}$	***h***^**2**^	SE	Single-trait			Multi-trait			
				Significant SNP	-log (***p***-value)^**a**^	Mean ***χ***^**2**^	Significant SNP^**a**^	-log (***p-***value)^**a**^	Mean ***χ***^**2**^	N GWAS equivalent
ADG	0.406	0.422	0.046	1	7.524	1.674	1359	14.581	1.815	1582
BWH	27,334.76	0.423	0.045	1	7.632	1.693	1209	14.392	1.781	1474
WW	10,937.59	0.386	0.046	1	7.454	1.718	1347	14.600	1.907	1654
HW	1658.12	0.450	0.045	0	5.706	1.727	1595	14.389	1.898	1617
HON	10,516.63	0.435	0.045	1	7.452	1.514	1138	13.005	1.694	1766
BLH	2.855	0.414	0.045	0	5.631	1.749	827	17.147	2.081	1890
FW	2951.46	0.343	0.045	1	7.592	1.541	833	17.750	1.649	1605
FY	0.0001	0.210	0.039	1	8.503	1.273	1920	11.622	1.335	1569

^a^For the most significant SNP; *ADG* average daily gain (g), *BWH* body weight at harvest (g), *WW* waste weight (g), *HW* head weight (g), *HON* gutted head-on weight (g), *BLH* body length at harvest (cm), *FW* fillet weight (g), *FY* fillet yield (%)

### Comparison between single-trait and multi-trait GWAS

The average gain in statistical power for mtGWAS compared to stGWAS was assessed by the increase in the mean *χ*^2^ statistic. Thus, we  calculated how much larger the stGWAS sample size would be expected, to be equivalent to the increase observed in *χ*^2^ statistic. We found that the mtGWAS analysis corresponded to gains equivalent to increase in the original sample size from 13 to 44%. These values corresponded to an increase in sample size from 1309 for stGWAS to a value ranging from 1474 to 1890 for mtGWAS (Table 2). For instance, the number of SNP surpassing the Bonferroni corrected significance threshold for stGWAS and mtGWAS, respectively, was: 1 and 1359 for ADG, 1 and 1209 for BWH, 1 and 1347 for WW, 0 and 1595 for HW, 1 and 1138 for HON, 0 and 827 for BLH, 1 and 833 for FW, and 1 and 1920 for FY. In addition, the maximum -log(*p*-value) increased from 7.52 to 14.58 for ADG, from 7.63 to 14.39 for BWH, from 7.45 to 14.60 for WW, from 5.71 to 14.39 for HW, from 7.45 to 13.00 for HON, from 5.63 to 17.15 for BLH, from 7.59 to 17.75 for FW, and from 8.50 to 11.62 for FY, when comparing stGWAS against mtGWAS (Table 2).

**Fig. 2:**
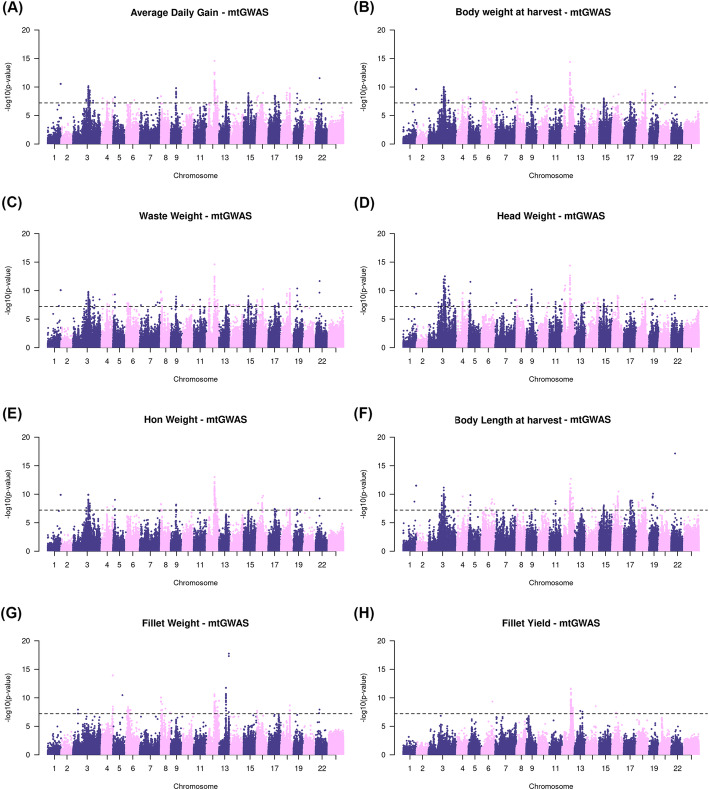
Manhattan plot for multi-trait GWAS (mtGWAS) for eight body traits in Nile tilapia. Manhattan plots of SNPs associated with: **a** Average daily gain. **b** Body weight at harvest. **c** Waste weight. **d** Head weight. **e** Gutted head-on weight. **f** Body length at harvest. **g** Fillet weight. **h** Fillet yield. The x-axis presents genomic coordinates along chromosomes 1–23 in Nile tilapia. On the y-axis the negative logarithm of the SNPs associated *p-*value is displayed. The dashed black line represents the genome-wide significance threshold after Bonferroni correction (−log_10_ (*p-*value > 7.21e-8)

The stGWAS identified a single significant genomic region on LG16, in position 4,178,535 base pairs (bp), associated with ADG, BWH, WW, HON and FW, and a significant SNP on LG07, in position 16,847,179 bp, for FY (Supplementary Fig. 1). When combining the summary statistics of all body traits, using mtGWAS, we identified several novel genomic regions associated with different traits. The number of SNPs surpassing the genome-wide significance threshold ranged from 827 to 1920 depending on the trait analyzed, with the lowest and the highest number of significant variants associated with BLH and FW (Table 3). The greatest number of significant variants were located on LG03 and LG12 for all traits, except FW where most of the variants were located on LG13 (Fig. 2). The location of significant variants on different chromosomes, and representation of several loci, suggest that these body traits are under polygenic control.

**Table 3:** Genomic regions and the closest candidate genes for the top five lead SNPs associated with body traits based on multi-trait GWAS in Nile tilapia

Marker^a^	LG^b^	Position^c^	Alleles	MAF^d^	*p-*value	Closest genes^e^
Average daily gain						
12:24557870	12	24,557,870	[A/G]	0.069	2.627E-15	HSD17B4, SEMA6A
12:24557984	12	24,557,984	[T/C]	0.069	2.627E-15	HSD17B4, SEMA6A
22:11998439	22	11,998,439	[G/A]	0.059	2.800E-12	DPY19L4, GDF6
1:39153024	1	39,153,024	[G/A]	0.052	2.821E-11	CCDC102A, HDGFL3
1:39193509	1	39,193,509	[A/G]	0.052	2.821E-11	uncharacterized
Body weight at harvest						
12:24557870	12	24,557,870	[A/G]	0.069	4.055E-15	HSD17B4, SEMA6A
12:24557984	12	24,557,984	[T/C]	0.069	4.055E-15	HSD17B4, SEMA6A
1:39153024	1	39,153,024	[G/A]	0.052	2.444E-10	CCDC102A, HDGFL3
1:39193509	1	39,193,509	[A/G]	0.052	2.444E-10	uncharacterized
1:39558113	1	39,558,113	[G/A]	0.052	2.444E-10	ZNF536, CCNE1
Waste weight						
12:24557870	12	24,557,870	[A/G]	0.069	2.512E-15	HSD17B4, SEMA6A
12:24557984	12	24,557,984	[T/C]	0.069	2.512E-15	HSD17B4, SEMA6A
22:11998439	22	11,998,439	[G/A]	0.059	2.130E-12	DPY19L4, GDF6, KDM1B
16:20105934	16	20,105,934	[G/A]	0.053	5.481E-11	MYO16, IRS2
16:20116545	16	20,116,545	[T/C]	0.053	5.481E-11	MYO16, IRS2, COL4A1
Head weight						
12:24557870	12	24,557,870	[A/G]	0.069	4.084E-15	HSD17B4, SEMA6A
12:24557984	12	24,557,984	[T/C]	0.069	4.084E-15	HSD17B4, SEMA6A
3:50439330	3	50,439,330	[C/T]	0.109	3.113E-13	uncharacterized
3:50439365	3	50,439,365	[T/C]	0.109	3.113E-13	uncharacterized
4:17899270	4	17,899,270	[G/T]	0.051	2.454E-10	uncharacterized
Gutted head-on weight						
12:24557870	12	24,557,870	[A/G]	0.069	9.893E-14	HSD17B4, SEMA6A
12:24557984	12	24,557,984	[T/C]	0.069	9.893E-14	HSD17B4, SEMA6A
3:47137003	3	47,137,003	[A/C]	0.110	1.170E-10	TLR2
1:39153024	1	39,153,024	[G/A]	0.052	1.278E-10	CCDC102A, HDGFL3
1:39193509	1	39,193,509	[A/G]	0.052	1.278E-10	uncharacterized
Body length at harvest						
22:11998439	22	11,998,439	[G/A]	0.059	7.129E-18	DPY19L4, GDF6, KDM1B
12:27146675	12	27,146,675	[C/T]	0.079	1.956E-13	GPX8, MCIDAS, ISCA1
1:39153024	1	39,153,024	[G/A]	0.052	3.146E-12	CCDC102A, HDGFL3
1:39193509	1	39,193,509	[A/G]	0.052	3.146E-12	CCDC102A, HDGFL3
1:39558113	1	39,558,113	[G/A]	0.052	3.146E-12	CCNE1, ZNF536
Fillet weight						
13:30002073	13	30,002,073	[A/G]	0.174	1.778E-18	uncharacterized
4:34954382	4	34,954,382	[T/C]	0.107	1.193E-14	SAMD14, PSMD3
4:34954397	4	34,954,397	[A/G]	0.107	1.193E-14	SAMD14, PSMD3
4:34958811	4	34,958,811	[A/G]	0.107	1.193E-14	SAMD14, PSMD3
4:34958990	4	34,958,990	[G/A]	0.107	1.193E-14	SAMD14, PSMD3
Fillet yield						
12:26984411	12	26,984,411	[G/A]	0.066	2.388E-12	uncharacterized
6:33824877	6	33,824,877	[T/G]	0.055	4.496E-10	XYLT1, RPS15A, COQ7
14:30148797	14	30,148,797	[G/T]	0.056	3.015E-09	uncharacterized
13:17730096	13	17,730,096	[C/A]	0.113	2.012E-08	MARCH8
13:17730605	13	17,730,605	[C/A]	0.113	2.012E-08	MARCH8

^a^Markers in bold indicate a common lead SNP in at least two traits

^b^Linkage group

^c^Position in base pairs

^d^Minor allele frequency

^e^Based on O_niloticus_UMD_NMBU as reference genome for *Oreochromis niloticus.* The full list of lead SNPs is available in S2 Table

Most of the lead SNPs were on LG01, LG03 and LG12 for ADG, BWH, WW, HW, HON and BLH. Some variants were common between body traits, such as two SNPs at positions 24,557,870 and 24,557,984 on LG12, that were the most significant SNPs (*p*-value < 9.893E-14) common in ADG, BWH, WW, HW, and HON. The lead SNPs for FW and FY were found on LG04 and LG13, and none of those were identified in other body traits (Table 3)*.*

### Candidate genes

The full list of genes located within 100 kb upstream and downstream of the lead SNP is available in additional file (Supplementary Table 2). Some lead SNPs for ADG, BWH, WW, HON, BLH are close to candidate genes, including *collagen type IV alpha 1 chain* (*COL4A1*) and *growth differentiation factor 6* (*GDF6*) on LG16 and LG22, respectively, and *ankyrin repeat and SOCS box containing 2* (*ASB2*) associated with BWH and HON, located on LG19. The genes intercepted by lead SNPs, located in exonic or intronic regions are shown in Table 4. Some of these genes have been associated with body traits in previous studies. For FW, the gene *A disintegrin and metalloproteinase with thrombospondin motifs 9* (*ADAMTS9*), located in LG05, was intercepted by a SNP in an exon region at 29,062,243 bp. Two lead SNPs for WW, located on LG09, at positions 14,670,077 and 14,674,835 bp, intercepted introns of the gene *solute carrier family 4 member 2* (SLC4A2). Intronic regions of *α1,6-fucosyltransferase* (*FUT8*) and the *heart development protein with EGF like domains 1* (*HEG1*), located on LG15 and LG16, were intercepted by lead SNPs associated with ADG and FY, respectively. Two SNPs within *nucleoporin 107* (*NUP107*), located on LG17, were associated with both BWH and HON, on positions 19,609,147 and 19,612,729 bp, respectively, with the first SNP hitting an intronic region and the second one located in an exon region. Others genes such as *Coiled-Coil Domain Containing 102A* (*CCDC102A*), *SLIT-ROBO Rho GTPase Activating Protein 1* (*SRGAP1*), *MutS Homolog 6* (*MSH6*) *Myosin VI* (*MYO6*), *Myosin XVI* (*MYO16*), and *Kinectin 1* (*KTN1*) were intercepted by one or more lead SNPs, but no clear evidence of a close association with body size and growth-related traits has been reported.

**Table 4:** Genes intercepted by a lead SNP associated with body traits based on multi-trait GWAS in Nile tilapia

Gene^a^	LG^b^	Position^c^	N SNP^d^	*p-*values^e^		Genomic location	Traits
				Min	Max		
CCDC102A	1	39,153,024	1	3.146E-12	3.474E-10	Intronic	ADG, BWH, WW, HW, HON, BLH
ADAMTS9	5	29,062,243	1	3.446E-11	–	Exonic	FW
SRGAP1	7	60,999,336–61,005,198	3	4.614E-09	4.614E-09	Intronic	HW
SLC4A2	9	14,670,077–14,674,835	2	5.225E-08	5.225E-08	Intronic	WW
MALRD1	9	16,267,509–16,328,834	2	1.678E-10	5.325E-08	Intronic	ADG, BWH, HON
PTPRN2	9	16,433,765–16,435,917	2	4.091E-09	4.758E-09	Intronic	ADG, HW
DMXL1	12	24,525,556	1	2.379E-11	–	Intronic	FW
MARCH8	13	17,730,096–17,730,605	1	2.012E-08	2.012E-08	Intronic	FY
MSH6	13	21,626,153–21,626,426	1	3.796E-08	3.796E-08	Exonic/Intronic	ADG
FUT8	15	14,457,958	1	4.861E-08	–	Intronic	ADG
TMEM121	15	14,662,118	1	9.425E-10	9.844E-09	Intronic	ADG, BWH, WW
MYO6	15	23,976,527	1	5.175E-08	–	Intronic	HON
HEG1	16	12,574,352	1	3.836E-08	–	Intronic	FY
DOCK9	16	17,284,162	1	1.673E-08	–	Intronic	HW
MYO16	16	20,105,934–20,116,545	2	3.340E-11	1.988E-09	Intronic/Exonic	ADG, BWH, WW, HON, BLH
NUP107	17	19,609,147–19,612,729	2	3.815E-08	4.102E-08	Intronic/Exonic	BWH, HON
KTN1	19	11,094,375	1	7.954E-09	–	Exonic	ADG

*ADG* average daily gain (g), *BWH* body weight at harvest (g), *WW* waste weight (g), *HW* head weight (g), *HON* gutted head-on weight (g), *BLH* Body length at harvest (cm), *FW* fillet weight (g), *FY* fillet yield (%)

^a^Genes intercepted by at least one lead SNP based on O_niloticus_UMD_NMBU as reference genome for *Oreochromis niloticus*

^b^Linkage group

^c^In base pairs

^d^Number of lead SNPs

^e^Minimum (Min) and maximum (Max) *p-*value for coincident lead SNP for at least two traits

## Discussion

We found moderate to high heritability values for ADG, BWH, WW, HW, HON, BLH, FW and FY, which is consistent with previous estimates for Nile tilapia calculated using pedigree and genomic methods [8, 9, 20, 21]. The additive genetic variance and heritability estimated for BWH using genotypes imputed to high-density genotypes increased about 15% in comparison to the value previously estimated for the same population using a 50 K SNP panel [20].

The use of genomic information can help in the identification of QTLs controlling complex traits which are economically important for aquaculture purposes, such as growth-related traits. Previous studies have identified loci and candidate genes associated with growth-related traits in aquaculture species [20, 23, 24, 26, 37, 38]. However, similar to what we found when using stGWAS (Supplementary Fig. 1), few or no markers surpassed the genome-wide significance threshold, or represented a small proportion of genetic variance for all body traits studied here. No studies have found evidence of major QTLs for growth-related traits, and GWAS signals were moderate even when a relatively large sample size (> 4600 animals) and more than 100 K markers were used, as in the case of GWAS for body weight in Atlantic salmon [23].

To increase the statistical power, in order to detect genetic association between SNPs and traits of interest, recent studies have used mtGWAS, which can leverage multiple summary statistics from GWAS performed on the same trait with different measures or different traits with a high genetic correlation among them [33, 39, 40]. We combined the use of genotypes imputed to high-density and the mtGWAS approach implemented in MTAG software to increase the statistical power and accuracy of QTL detection [33]. The imputation proceeded from a medium-density (50 K) SNP panel to high-density, where the markers from the reference dataset were previously selected based on quality control, and an expected accuracy of imputation higher than 0.80. The mtGWAS increases statistical power by using information from different traits that are genetically correlated with each other [33]. Here, the correlation of the overall SNP effects ranged from 0.86 to 1.00, except for the correlation between FY and all of the other traits, which ranged from 0.20 to 0.47 (Fig. 1), and the samples were overlapped for all traits. The better resolution of the genotypes imputed to high-density, combined with the power of the mtGWAS approach, lead to the detection of several novel significant markers not previously found when using stGWAS.

A difference in the number of significant SNPs between stGWAS and mtGWAS is expected given the substantial increase in statistical power which has been documented for the mtGWAS approach. However, it has also been shown that original associations detected by single-trait GWAS can disappear when running multi-trait GWAS. For instance, in the paper describing the application of mtGWAS [33], the increase of significant lead SNPs was from two up to four times higher when comparing mtGWAS against stGWAS. Nevertheless, there were also SNPs associated in the stGWAS analyses which were not found to be associated when running a multi-trait GWAS. If the SNP association is not confirmed by the mtGWAS, we may assume that the previous association identified by the stGWAS is spurious and interpretations on these unconfirmed associations have to be taken with caution.

We found numerous significant markers associated with body traits, dispersed in almost all linkage groups (LG; Fig. 2), probably due to the polygenic architecture of these traits in Nile tilapia. However, a major common association peak on LG12 was found for all traits analyzed, except for FW where the major peak was found on LG13; suggesting that part of the genetic variation that affects body traits might be explained by loci on these linkage groups. No gene was intercepted by the two most significant lead SNPs in this region, but a nearby gene on LG12, *hydhroxysteroid 17-Beta Dehydrogenase 4 (HSD17B4)*, a possible regulator of muscle development in Berkshire pigs*,* has been reported to play an important role during the early stages of myogenesis when the expression of its mRNA is significantly high [41].

Some lead SNPs identified in this study were located close or intercepted several strong functional candidate genes associated with body and growth-related traits in previous studies. For instance, strong functional candidate genes were found in windows within 100 kb downstream and upstream from the lead SNP, such as *COL4AI,* located in LG16*,* associated with different body traits, including ADG, BWH, WW, HON and BLH. In catfish *COL4A1* was identified within QTLs associated with body length and body length of the fish without the head. Collagen is an important component of the extracellular matrix of cartilage and bone, playing a key role in skeletal development [42]. We also found *GDF6,* located in LG22*,* which was associated with different traits including ADG, BWH, WW, HON, BLH. Mutations in *GDF6* in zebrafish is related with reduced eye size and different skeletal defects [43]. In a study aimed to compare the orthologous sequences from 14 species (including human, mice, livestock, fugu, and zebrafish), the *GDF6* gene was found to control developmental patterning of skeletal joints [44]. Inactivation of the *GDF6* gene can cause defects in the joints, ligaments, and cartilage formation in mouse [45]. In addition, the *ASB2* gene, located in chromosome 19, was associated with BWH and HON. In Atlantic salmon, the *ASB2* gene is not involved in muscle differentiation but may play an important role in growth inhibition. The high expression of *ASB2* observed in skeletal muscle of fasting fish is strongly downregulated in response to feeding [46].

We also found strong candidate genes intercepted by lead SNPs that may contribute to a better understanding of the biological mechanisms controlling body traits in Nile tilapia. Growth is considered a continuous function during the life of an animal and ADG is an important trait which can be targeted to select for rapid growth. ADG was previously reported as a selection criteria in a breeding program for Nile tilapia, which has applied selection for at least five generations [47]. We found a lead SNP associated with ADG on LG15, located in an intronic region of the *FUT8* gene, which has been associated with ADG (from birth to six months-age) in a sheep population from Iran [48]. In mice, the disruption of the *FUT8* gene induces severe growth retardation and early mortality during postnatal development [49–52]. The *insulin-like growth factor binding protein-3* (*IGFBP-3*) has growth inhibitory effects, and the alteration in the function of *low-density lipoprotein receptor-related protein-1* (*LRP-1*) is a result of the loss of core fucosylation that might cause an elevated serum concentration of *IGFBP-3* in *FUT8*-null mice [52]. The loss of function of *FUT8* has also been reported to be related to down-regulation of *transforming growth factor-beta 1* (*TGF-β1*) *receptor* and *epidermal growth factor* (*EGF*) receptor, proteinase-activated receptor and integrin activity, which contributes to emphysema-like changes in the lung, and growth retardation in *FUT8*-null mice [51].

Two lead SNPs associated with BWH and HON were found on LG17, in an intronic and exonic region of the *NUP107* gene which plays an important role in the development of vertebrate embryos. The zygotic deficiency of *NUP107* in zebrafish embryos can result in loss of pharyngeal skeletons, degeneration of intestinal and retinal epithelia, and implications in cartilage and bone formation [53]. In senescent fibroblasts of humans and organs of aged mice, a decreased level of *NUP107* is suggested to be involved in the hypo-responsiveness to growth [54].

The waste weight is the sum of the weight of the head, viscera, bones and fins, and has been suggested as an alternative phenotypic record to improve fillet yield through the application of various index (e.g. fillet to waste ratio). However, based on simulated data of ten generations of selection using real genetic parameters of five farmed fish populations, direct selection on fillet yield was generally the best approach to improve the trait [13]. The potential limitation for selecting against waste weight is the probability of decreasing the volume of essential organs in the visceral cavity. A negative genetic correlation (< − 0.52) between fillet yield and head, and bone development has been reported in rainbow trout [55]. We found a lead SNP that intercepts the *SLC4A2* gene, a strong biological candidate for waste weight in Nile tilapia. The loss of function of this gene causes emaciation and achlorhydric condition [56], generating severe growth retardation, reduced osteoclast numbers and/or a reduction in osteoclast activity, resulting in osteopetrosis in mice [57]. Osteopetrosis is a skeletal disorder that can affect humans and animals, characterized by the formation of overly dense bones [57, 58]. In Red Angus cattle, a deletion mutation in *SLC4A2* is associated with an osteopetrosis phenotype [58].

Fillet traits are key economic characteristics for aquaculture species and new insights regarding the underlying genetic variants controlling them can help to enhance yield. We found two lead SNPs associated with FW and FY, intercepting an exonic and intronic region of genes *ADAMTS9* and *HEG1*, respectively. The *ADAMTS9* gene is highly expressed during embryo development and continues to be expressed in adult tissues of mice [59, 60]. A significant expression of *ADAMTS9* during skeletal development of mouse was suggested by Jungers et al. (2005), including mandible, ossification centers, initial condensation of mesenchyme to form the cartilage centers, perichondrium around formed cartilage, proliferative zones of cartilage and long bones. Skeletal development may be correlated with organic growth [61]. *ADAMTS9* is responsible for the regulation of the *epidermal growth factor receptor* (*EGFR*) and *TGF-β1* [62, 63]. Tang et al. (2019) [64] identified a 22-bp indel in *ADAMTS9* associated with chest width, chest width index and chest circumference index, and a14-bp indel associated with height across the hip in cashmere goats, which suggests that *ADAMTS9* is a functional molecular marker that can be used to improve growth traits in goats [62].

A lead SNP associated with FY intercepted the *HEG1* gene, located in LG16. The *HEG1* gene was initially reported to be responsible for regulating zebrafish heart growth and the development of heart and blood vessels development [65]. Recently the *HEG1* gene was identified as one of several novel genes associated with human skeletal muscle growth, exhibiting a significant correlation with the percentage of change in lean mass [66, 67]. In a comparative transcriptomic analysis aimed to identify differentially expressed genes related to product performance and meat quality from the *longissimus dorsi* in sheep, Cheng et al. (2020) [68] identified six differentially expressed genes, including *HEG1* [66].

Some genes such as *MSH6, SRGAP1*, *MYO6*, *MYO16, KTN1* and other molecules presented in Table 4, are intercepted by lead SNPs and thought to be involved in different biological functions and conditions, such as some types of cancer [69, 70], hearing loss [71] and schizophrenia [72]. The mutation of *MSH6,* for example, may increase the risk of developing colorectal carcinomas [73, 74], and *MYO16* appears to have an important role in neural development and the function of the nervous system [75]. The functional relationship between these genes and the variation in growth-related traits in Nile tilapia is unclear. Thus,  the function of the identified genes and their potential relationship with body traits in Nile tilapia must be better characterized.

## Conclusions

We used dense genotypic information to refine association mapping analysis for body traits in Nile tilapia and found that mtGWAS provided substantial improvements in the number of significant SNPs identified when compared to stGWAS. These results confirm the increase of statistical power to identify trait-specific genetic associations in multi-trait analysis. Interestingly, we found several lead SNPs within or nearby genes related to cartilage, bone, skeletal growth and development in humans, mice, livestock and aquaculture species. These results can provide further knowledge and a better understanding of genetic variants and genes underlying complex body traits in Nile tilapia.

## Material and methods

### Animals and phenotypes

We used a total of 1309 phenotyped animals from 72 families (mean = 18, minimum = 7, and maximum = 25 animals per family) belonging to a breeding nucleus owned by Aquacorporación International group (ACI), Costa Rica. More details about the breeding program, the origin of the Nile tilapia population and production conditions are described in detail in previous studies [18, 20, 76]. Briefly, a mating design of two dams per sire was used to produce the 72 full-sib families. The eggs of each full-sib family were incubated and reared in separate hapas until individual tagging by using PIT (passive Integrated Transponder)-tags at an average weight and age of 13 g (SD = 8 g) and 104 days (SD = 18 days), respectively. After tagging, the fish were grown in excavated ponds for about 370 days until harvest. All animals were slaughtered by hypothermia in ice slurry at commercial processing plant, and different body traits were measured at harvest time: body weight at harvest (BWH in g), fillet weight (FW in g), waste weight (WW in g = BWH – FW), head weight (HW in g), gutted head-on weight (HON in g = BWH – Viscera), body length at harvest (BLH in cm), average daily gain (ADG in g = (BWH - body weight at tagging)/(age at harvest - age at tagging)), and fillet yield (FY in % = FW/BHW*100).

### Genotypes and imputation to whole-genome sequences

Genomic DNA was extracted and purified from 1309 fin clip samples using the DNeasy Blood & Tissue Kit (QIAGEN) according to the manufacturer’s protocol. The samples were genotyped using a 50 K SNP Illumina BeadChip [18] and filtered using departure from Hardy-Weinberg Equilibrium (HWE, *p*-value 10^− 6^), minor allele frequency (MAF < 0.01), and a genotyping call-rate for SNPs and samples of < 0.90. After quality control 29,587 SNPs and 1309 samples were retained.

Initially, 26.6 million non-redundant SNP variants were identified through Illumina HiSeq 2500 re-sequencing performed in 143 animals from the breeding nucleus owned by Aquacorporación International group (ACI), Costa Rica [18]. Quality control of WGS genotypes was performed using the following thresholds: HWE (*p-*value 10^− 8^), MAF < 0.01 and call-rate for SNPs < 0.80. A total of 5,011,051 SNPs were retained after applying the filters described above. In order to estimate the overall accuracy of imputation and remove the variants with low imputation accuracy we used a five-fold cross validation scheme. Briefly, the 143 animals with data from the WGS-derived genotypes were randomly divided into five exclusive reference sets (80% of animals genotyped with ~ 5 million SNPs) and the remaining animals were used as the validation set (20% of animals with medium-density genotypes). The accuracy of imputation was estimated as the correlation between true and imputed genotypes (R^2^ value). A total of 1,324,420 SNPs with R^2^ value higher than 0.80 were used as the final ultra-dense SNP panel for imputation. The 143 re-sequenced animals and 1,324,420 SNPs were used as a reference dataset to impute the 1309 animals with medium-density SNP genotypes using the software FImpute v. 3.0 [77]. A post-imputation quality control excluded SNPs with MAF < 0.05 and HWE *p*-value < 10^− 8^, resulting in a total of 992,494 SNPs available for downstream analyses.

### Single-trait genome-wide association

The single-trait genome wide association analyses (stGWAS) were performed using the *mlma* option of the software GCTA v. 1.24 [78], which was used to apply the following linear mixed model:

1 $$ {\mathrm{y}}_{\mathrm{i}\mathrm{j}}=\upmu +{\mathrm{b}}_1\ast {\mathrm{a}\mathrm{ge}}_{\mathrm{j}}+{\mathrm{b}}_2\ast {\mathrm{SNP}}_{\mathrm{i}}+{\mathrm{a}}_{\mathrm{i}\mathrm{j}}+{\mathrm{e}}_{\mathrm{i}\mathrm{j}} $$

where *y*_*ij*_ is the phenotypic value of the *j-*th animal, μ is the fixed effect of the overall mean, *b*_1_ and *b*_2_ are the regression coefficients for age and the allele substitution effect for SNP, respectively, *age*_*j*_ is the age covariate of the *j-th* animal and *SNP*_*i*_ is the *i-*th SNP genotype of animal *j*, coded as 0, 1 and 2 for genotype A_1_A_1_, A_1_A_2_ and A_2_A_2_, respectively, *a*_*ij*_ is the random polygenic effect of the *j-*th animal $\sim \mathrm{N}\left(0,\mathbf{G}{\upsigma}_{\mathrm{a}}^2\right)$, with **G** representing the genomic relationship matrix (GRM) calculated using the imputed genotypes and ${\upsigma}_{\mathrm{a}}^2$ the genetic variance [78, 79], and *e*_*ij*_ is the random residual effect $\sim \mathrm{N}\left(0,\mathbf{I}{\upsigma}_{\mathrm{e}}^2\right)$, with **I** representing an identity matrix and ${\upsigma}_{\mathrm{e}}^2$ the residual variance. The GRM is calculated based on the relationship from a genome-wide sample of SNPs obtained by using a common-sense weighting scheme [78]. The GRM restricted maximum likelihood (GREML) [78] implemented in GCTA was used to estimate the genetic and residual variances. Heritability (h^2^) was calculated as h^2^ = ${\upsigma}_{\mathrm{a}}^2$ /$\left({\upsigma}_{\mathrm{a}}^2+{\upsigma}_{\mathrm{e}}^2\right).$ For each SNP, the allele substitution effect and its *p*-value were also estimated using GCTA.

### Multi-trait genome-wide association

The summary statistics from stGWAS were used as input for the multi-trait analysis of GWAS (mtGWAS) performed using the software MTAG v0.9.0 [33]. In MTAG, the SNP effect estimated for each trait can be improved when different traits that are correlated are included in the analysis. This multi-trait approach can increase the power to detect loci in any of the traits assessed. The first step of MTAG is to filter variants based on discarding non common SNPs, duplicated SNPs, or SNPs with strand ambiguity. In our study, out of the 992,494 SNPs available after imputation and initial quality control, a total of 183,401 SNPs with strand ambiguity were filtered out. The remaining 809,093 SNPs were used for mtGWAS analyses. A bivariate linkage disequilibrium (LD) score regression was used, thus summary statistics do not need to come from independent samples [33]. The MTAG output consists of a file per trait with updated results of SNP effects and *p*-values from a mtGWAS, which can be interpreted in the same way as stGWAS. Significance thresholds were determined for both single-trait and mtGWAS using Bonferroni correction (0.05/ number of SNPs).

To calculate how much larger the stGWAS sample size would have to be to give the same mean χ^2^ statistics than mtGWAS, the following equation was used [33]:

2 $$ {\mathrm{N}}_{\mathrm{GWAS}\ \mathrm{equivalent}}={\mathrm{N}}_{\mathrm{GWAS}}\frac{\overline{\upchi_{\mathrm{mtGWAS}}^2}}{\overline{\upchi_{\mathrm{stGWAS}}^2}} $$

where, ${\upchi}_{\mathrm{mtGWAS}}^2$ and ${\upchi}_{\mathrm{stGWAS}}^2$ are the mean *χ*^2^ statistic for mtGWAS and stGWAS results, respectively, and N_GWAS_ is the number of actual sample size in stGWAS (1309 animals).

### Identification of QTL and candidate genes

The most significant SNP per chromosome per each trait, detected using mtGWAS, was selected as the lead SNP and furtherly used to search for candidate genes based on proximity to the variant. Genes located within 100 kb upstream and downstream of the lead SNP were considered putative candidate genes associated with the trait. The gene search was performed using BLAST (Basic Local Alignment Search Tool) against the latest version of the *Oreochromis niloticus* reference genome (O_niloticus_UMD_NMBU [15]), which is publicly available at NCBI (GenBank assembly accession GCA_001858045.3).

## Supplementary Information

**Additional file 1: Supplementary Fig. 1.** Manhattan plot for single-trait GWAS (stGWAS) for body traits in Nile tilapia. Manhattan plots of SNPs associated with: (A) Average daily gain. (B) Body weight at harvest. (C) Waste weight. (D) Head weight. (E) Gutted head-on weight. (F) Body length at harvest. (G) Fillet weight. (H) Fillet yield. The x-axis presents genomic coordinates along chromosomes 1–23 in Nile tilapia. On the y-axis the negative logarithm of the SNPs associated *p*-value is displayed. The dashed black line represents the genome-wide significance threshold after Bonferroni correction (−log_10_ (*p-*value > 7.30e-8)).

**Additional file 2: Supplementary Table 1.** Summary results from genotype quality control of whole-genome sequence (WGS), imputed WGS genotypes, and 50 K single nucleotide polymorphism (SNP) chip for Nile tilapia.

**Additional file 3: Supplementary Table 2.** Genomic regions and candidate genes for all lead SNPs associated with body traits based on multi-trait GWAS for Nile tilapia

## Data Availability

The datasets generated and/or analysed during the current study are available in the Figshare repository, https://figshare.com/s/9b265a22b7e138c5a839 and https://figshare.com/s/1fa22386fd5bae0366e0. The *Oreochromis niloticus* reference genome is publicly available at NCBI (GenBank assembly accession GCA_001858045.3, https://www.ncbi.nlm.nih.gov/genome/?term=GCA_001858045.3).

## Abbreviations

*ADAM12*: *Disintegrin and metalloproteinase domain 12*
*ADAMTS9*: *A Disintegrin and metalloproteinase with thrombospondin motifs 9*
ADG: Average daily gain
*ASB2*: *Ankyrin repeat and SOCS box containing 2*
BLAST: Basic Local Alignment Search Tool
BLH: Body length at harvest
bp: Base pairs
BWH: Body weight at harvest
*CCDC102A*: *Coiled-Coil Domain Containing 102A*
*COL4A1*: *Collagen type IV alpha 1 chain*
*EGF*: *Epidermal growth factor*
*EGFR*: *Epidermal growth factor receptor*
*FGF*: *Fibroblast growth factors*
*FUT8*: *α1,6-fucosyltransferase*
FW: Fillet weight
FY: Fillet yield
*GDF6*: *Growth differentiation factor 6*
GREML: Genomic-relationship matrix restricted maximum likelihood
GRM: Genomic-relationship matrix
GWAS: Genome-wide association studies
*HEG1*: *Heart development protein with EGF like domains 1*
HON: Gutted head-on weight
*HSD17B4*: *Hydroxysteroid 17-Beta Dehydrogenase 4*
HW: Head weight
HWE: Hardy-Weinberg equilibrium
*IGFBP-3*: *Insulin-like growth factor binding protein-3*
*KTN1*: *Kinectin 1*
LD: Linkage disequilibrium
LG: Linkage group
*LRP-1*: *Low-density lipoprotein receptor-related protein-1*
MAF: Minor allele frequency
MAS: Marker-assisted selection
*MEP1A*: *Meprin A subunit beta-like*
*MSH6*: *MutS Homolog 6*
mtGWAS: Multi-trait genome-wide association study
*MYLK*: *Myosin light chain kinase*
*MYO16*: *Myosin XVI*
*MYO6*: *Myosin VI*
*NUP107*: *Nucleoporin 107*
QTL: Quantitative trait loci
*SLC4A2*: *Solute carrier family 4 member 2*
SNP: Single nucleotide polymorphisms
*SRGAP1*: *SLIT-ROBO Rho GTPase Activating Protein 1*
stGWAS: Single-trait genome-wide association studies
*TGFBR3*: *Transforming growth-factor beta receptor type 3*
*TGF-β1*: *Transforming growth factor-beta 1*
WGS: Whole-genome sequence
WW: Waste weight

## References

[1] FAO (2019). Fisheries and aquaculture software. FishStatJ - Software for Fishery and Aquaculture Statistical Time Series.

[2] Gjedrem T (2012). Genetic improvement for the development of efficient global aquaculture: a personal opinion review. Aquaculture..

[3] Bentsen HB, Gjerde B, Nguyen NH, Rye M, Ponzoni RW, Palada de Vera MS (2012). Genetic improvement of farmed tilapias: Genetic parameters for body weight at harvest in Nile tilapia (*Oreochromis niloticus*) during five generations of testing in multiple environments. Aquaculture.

[4] Khaw HL, Bovenhuis H, Ponzoni RW, Rezk MA, Charo-Karisa H, Komen H (2009). Genetic analysis of Nile tilapia (Oreochromis niloticus) selection line reared in two input environments. Aquaculture..

[5] Ponzoni RW, Hamzah A, Tan S, Kamaruzzaman N (2005). Genetic parameters and response to selection for live weight in the GIFT strain of Nile Tilapia (Oreochromis niloticus). Aquaculture..

[6] Rezk MA, Ponzoni RW, Khaw HL, Kamel E, Dawood T, John G (2009). Selective breeding for increased body weight in a synthetic breed of Egyptian Nile tilapia, Oreochromis niloticus: response to selection and genetic parameters. Aquaculture..

[7] Rutten MJM, Bovenhuis H, Komen H (2005). Genetic parameters for fillet traits and body measurements in Nile tilapia (Oreochromis niloticus L.). Aquaculture..

[8] Rye M, Wang Y-X, Yang K-S, Bentsen HB, Gjedrem T, Thodesen (Da-Yong Ma) J (2011). Genetic improvement of tilapias in China: Genetic parameters and selection responses in growth of Nile tilapia (*Oreochromis niloticus*) after six generations of multi-trait selection for growth and fillet yield. Aquaculture.

[9] Nguyen NH, Ponzoni RW, Abu-Bakar KR, Hamzah A, Khaw HL, Yee HY (2010). Correlated response in fillet weight and yield to selection for increased harvest weight in genetically improved farmed tilapia (GIFT strain), *Oreochromis niloticus*. Aquaculture.

[10] Gjerde B, Mengistu SB, Ødegård J, Johansen H, Altamirano DS (2012). Quantitative genetics of body weight, fillet weight and fillet yield in Nile tilapia (Oreochromis niloticus). Aquaculture..

[11] Powell J, White I, Guy D, Brotherstone S (2008). Genetic parameters of production traits in Atlantic salmon (Salmo salar). Aquaculture..

[12] Yáñez JM, Joshi R, Yoshida GM. Genomics to accelerate genetic improvement in tilapia. Anim Genet. 2020:age.12989. 10.1111/age.12989. 10.1111/age.12989 32761644

[13] Fraslin C, Dupont-Nivet M, Haffray P, Bestin A, Vandeputte M (2018). How to genetically increase fillet yield in fish: new insights from simulations based on field data. Aquaculture..

[14] Bosworth B, Waldbieser G, Garcia A, Tsuruta S, Lourenco D (2020). Heritability and response to selection for carcass weight and growth in the Delta select strain of channel catfish, *Ictalurus punctatus*. Aquaculture.

[15] Conte MA, Joshi R, Moore EC, Nandamuri SP, Gammerdinger WJ, Roberts RB (2019). Chromosome-scale assemblies reveal the structural evolution of African cichlid genomes. Gigascience.

[16] Lu S, Zhu J, Du X, Sun S, Meng L, Liu S (2020). Genomic selection for resistance to Streptococcus agalactiae in GIFT strain of Oreochromis niloticus by GBLUP, wGBLUP, and BayesCπ. Aquaculture..

[17] Cáceres G, López ME, Cádiz MI, Yoshida GM, Jedlicki AM, Palma-Véjares R (2019). Fine Mapping Using Whole-Genome Sequencing Confirms Anti-Müllerian Hormone as a Major Gene for Sex Determination in Farmed Nile Tilapia (*Oreochromis niloticus* L.). G3 (Bethesda).

[18] Yáñez JM, Yoshida G, Barria A, Palma-Véjares R, Travisany D, Díaz D (2020). High-throughput single nucleotide polymorphism (SNP) discovery and validation through whole-genome Resequencing in Nile Tilapia (Oreochromis niloticus). Mar Biotechnol..

[19] Joshi R, Árnyasi M, Lien S, Gjøen HM, Alvarez AT, Kent M (2018). Development and Validation of 58K SNP-Array and High-Density Linkage Map in Nile Tilapia (*O. niloticus*). Front Genet.

[20] Yoshida G, Lhorente J, Correa K, Soto J, Salas D, Yáñez J (2019). Genome-Wide Association Study and Cost-Efficient Genomic Predictions for Growth and Fillet Yield in Nile Tilapia ( *Oreochromis niloticus* ). G3.

[21] Joshi R, Skaarud A, de Vera M, Alvarez AT, Ødegård J (2020). Genomic prediction for commercial traits using univariate and multivariate approaches in Nile tilapia (Oreochromis niloticus). Aquaculture..

[22] Bush WS, Moore JH. Chapter 11: genome-wide association studies. PLoS Comput Biol. 2012;8:e1002822. 10.1371/journal.pcbi.1002822 PMC3531285 23300413

[23] Yoshida GM, Lhorente JP, Carvalheiro R, Yáñez JM (2017). Bayesian genome-wide association analysis for body weight in farmed Atlantic salmon (*Salmo salar* L.). Anim Genet.

[24] Tsai HY, Hamilton A, Tinch AE, Guy DR, Gharbi K, Stear MJ (2015). Genome wide association and genomic prediction for growth traits in juvenile farmed Atlantic salmon using a high density SNP array. BMC Genomics.

[25] Geng X, Liu S, Yao J, Bao L, Zhang J, Li C (2016). A genome-wide association study identifies multiple regions associated with head size in catfish. G3 genes, genomes. Genet..

[26] Li N, Zhou T, Geng X, Jin Y, Wang X, Liu S (2018). Identification of novel genes significantly affecting growth in catfish through GWAS analysis. Mol Gen Genomics.

[27] Sanchez MP, Govignon-Gion A, Croiseau P, Fritz S, Hozé C, Miranda G (2017). Within-breed and multi-breed GWAS on imputed whole-genome sequence variants reveal candidate mutations affecting milk protein composition in dairy cattle. Genet Sel Evol.

[28] Van Binsbergen R, Bink MCAM, Calus MPL, Van Eeuwijk FA, Hayes BJ, Hulsegge I (2014). Accuracy of imputation to whole-genome sequence data in Holstein Friesian cattle. Genet Sel Evol.

[29] Wu P, Wang K, Zhou J, Chen D, Yang Q, Yang X (2019). GWAS on imputed whole-genome Resequencing from genotyping-by-sequencing data for farrowing interval of different parities in pigs. Front Genet.

[30] Van Den Berg S, Vandenplas J, Van Eeuwijk FA, Bouwman AC, Lopes MS, Veerkamp RF (2019). Imputation to whole-genome sequence using multiple pig populations and its use in genome-wide association studies. Genet Sel Evol.

[31] Al Kalaldeh M, Gibson J, Duijvesteijn N, Daetwyler HD, MacLeod I, Moghaddar N (2019). Using imputed whole-genome sequence data to improve the accuracy of genomic prediction for parasite resistance in Australian sheep. Genet Sel Evol.

[32] Crispim AC, Kelly MJ, Guimarães SEF, E Silva FF, Fortes MRS, Wenceslau RR, et al. Multi-trait GWAS and new candidate genes annotation for growth curve parameters in Brahman cattle. PLoS One. 2015;10:e0139906. 10.1371/journal.pone.0139906 PMC4622042 26445451

[33] Turley P, Walters RK, Maghzian O, Okbay A, Lee JJ, Fontana MA (2018). Multi-trait analysis of genome-wide association summary statistics using MTAG. Nat Genet.

[34] Lee JJ, Wedow R, Okbay A, Kong E, Maghzian O, Zacher M (2018). Gene discovery and polygenic prediction from a genome-wide association study of educational attainment in 1.1 million individuals. Nat Genet.

[35] Hill WD, Marioni RE, Maghzian O, Ritchie SJ, Hagenaars SP, McIntosh AM (2019). A combined analysis of genetically correlated traits identifies 187 loci and a role for neurogenesis and myelination in intelligence. Mol Psychiatry.

[36] Lam M, Trampush JW, Yu J, Knowles E, Davies G, Liewald DC (2017). Large-scale cognitive GWAS meta-analysis reveals tissue-specific neural expression and potential Nootropic drug targets. Cell Rep.

[37] Reis Neto RV, Yoshida GM, Lhorente JP, Yáñez JM. Genome-wide association analysis for body weight identifies candidate genes related to development and metabolism in rainbow trout (*Oncorhynchus mykiss*). Mol Gen Genomics. 2019:1–9. 10.1007/s00438-018-1518-2. 10.1007/s00438-018-1518-2 30635785

[38] Gutierrez AP, Yáñez JM, Fukui S, Swift B, Davidson WS (2015). Genome-wide association study (GWAS) for growth rate and age at sexual maturation in Atlantic Salmon (Salmo salar). PLoS One.

[39] Grove J, Ripke S, Als TD, Mattheisen M, Walters RK, Won H (2019). Identification of common genetic risk variants for autism spectrum disorder. Nat Genet.

[40] Han X, Gharahkhani P, Mitchell P, Liew G, Hewitt AW, MacGregor S (2020). Genome-wide meta-analysis identifies novel loci associated with age-related macular degeneration. J Hum Genet..

[41] Jo JL, Hwang JH, Kwon SG, Park DH, Kim TW, Kang DG, et al. Association between a non-synonymous HSD17B4 single nucleotide polymorphism and meat-quality traits in Berkshire pigs. Genet Mol Res. 2016;15. 10.4238/gmr15048970. 10.4238/gmr15048970 27819726

[42] Olsen BR, Reginato AM, Wang W (2000). Bone Development. Annu Rev Cell Dev Biol.

[43] den Hollander AI, Biyanwila J, Kovach P, Bardakjian T, Traboulsi EI, Ragge NK (2010). Genetic defects of GDF6 in the zebrafish out of sight mutant and in human eye developmental anomalies. BMC Genet.

[44] Portnoy ME, McDermott KJ, Antonellis A, Margulies EH, Prasad AB, Kingsley DM (2005). Detection of potential GDF6 regulatory elements by multispecies sequence comparisons and identification of a skeletal joint enhancer. Genomics..

[45] Settle SH, Rountree RB, Sinha A, Thacker A, Higgins K, Kingsley DM (2003). Multiple joint and skeletal patterning defects caused by single and double mutations in the mouse Gdf6 and Gdf5 genes. Dev Biol.

[46] Bower NI, Johnston IA (2010). Discovery and characterization of nutritionally regulated genes associated with muscle growth in Atlantic salmon. Physiol Genomics.

[47] de Oliveira CAL, Ribeiro RP, Yoshida GM, Kunita NM, Rizzato GS, de Oliveira SN (2016). Correlated changes in body shape after five generations of selection to improve growth rate in a breeding program for Nile tilapia Oreochromis niloticus in Brazil. J Appl Genet..

[48] Pasandideh M, Rahimi-Mianji G, Gholizadeh M (2018). A genome scan for quantitative trait loci affecting average daily gain and Kleiber ratio in Baluchi sheep. J Genet.

[49] Wang X, Inoue S, Gu J, Miyoshi E, Noda K, Li W (2005). Dysregulation of TGF-β1 receptor activation leads to abnormal lung development and emphysema-like phenotype in core fucose-deficient mice. Proc Natl Acad Sci U S A.

[50] Wang X, Gu J, Miyoshi E, Honke K, Taniguchi N (2006). Phenotype changes of Fut8 knockout mouse: Core Fucosylation is crucial for the function of growth factor receptor(s). Methods Enzymol.

[51] Wang X, Fukuda T, Li W, Gao C-X, Kondo A, Matsumoto A (2009). Requirement of Fut8 for the expression of vascular endothelial growth factor receptor-2: a new mechanism for the emphysema-like changes observed in Fut8-deficient mice. J Biochem.

[52] Lee SH, Takahashi M, Honke K, Miyoshi E, Osumi D, Sakiyama H (2006). Loss of core fucosylation of low-density lipoprotein receptor-related protein-1 impairs its function, leading to the upregulation of serum levels of insulin-like growth factor-binding protein 3 in Fut8−/− mice. J Biochem.

[53] Zheng X, Yang S, Han Y, Zhao X, Zhao L, Tian T (2012). Loss of zygotic NUP107 protein causes missing of pharyngeal skeleton and other tissue defects with impaired nuclear pore function in zebrafish embryos. J Biol Chem.

[54] Kim SY, Kang HT, Choi HR, Park SC (2010). Reduction of Nup107 attenuates the growth factor signaling in the senescent cells. Biochem Biophys Res Commun.

[55] Haffray P, Bugeon JÔ, Pincent C, Chapuis H, Mazeiraud E, Rossignol MN (2012). Negative genetic correlations between production traits and head or bony tissues in large all-female rainbow trout (Oncorhynchus mykiss). Aquaculture..

[56] Gawenis LR, Ledoussal C, Judd LM, Prasad V, Alper SL, Stuart-Tilley A (2004). Mice with a targeted disruption of the AE2 cl−/HCO 3- exchanger are achlorhydric. J Biol Chem.

[57] Wu J, Glimcher LH, Aliprantis AO (2008). HCO3−/cl- anion exchanger SLC4A2 is required for proper osteoclast differentiation and function. Proc Natl Acad Sci U S A.

[58] Meyers SN, McDaneld TG, Swist SL, Marron BM, Steffen DJ, O’Toole D (2010). A deletion mutation in bovine SLC4A2 is associated with osteopetrosis in red Angus cattle. BMC Genomics.

[59] Clark ME, Kelner GS, Turbeville LA, Boyer A, Arden KC, Maki RA (2000). ADAMTS9, a novel member of the ADAM-TS/metallospondin gene family. Genomics..

[60] Jungers KA, Le Goff C, Somerville RPT, Apte SS (2005). Adamts9 is widely expressed during mouse embryo development. Gene Expr Patterns.

[61] González-Cerón F, Rekaya R, Aggrey SE (2015). Genetic analysis of bone quality traits and growth in a random mating broiler population - ScienceDirect. Poult Sci.

[62] Shao B, Feng Y, Zhang H, Yu F, Li Q, Tan C (2018). The 3p14.2 tumour suppressor ADAMTS9 is inactivated by promoter CpG methylation and inhibits tumour cell growth in breast cancer. J Cell Mol Med.

[63] Wang Q, Hao R, Zhao X, Huang R, Zheng Z, Deng Y (2018). Identification of EGFR in pearl oyster (Pinctada fucata martensii) and correlation analysis of its expression and growth traits. Biosci Biotechnol Biochem.

[64] Tang Q, Zhang X, Wang X, Wang K, Yan H, Zhu H (2019). Detection of two insertion/deletions (indels) within the ADAMTS9 gene and their associations with growth traits in goat. Small Rumin Res.

[65] Kleaveland B, Zheng X, Liu JJ, Blum Y, Tung JJ, Zou Z (2009). Regulation of cardiovascular development and integrity by the heart of glass-cerebral cavernous malformation protein pathway. Nat Med.

[66] Seaborne RA, Strauss J, Cocks M, Shepherd S, O’Brien TD, Van Someren KA (2018). Human skeletal muscle possesses an epigenetic memory of hypertrophy. Sci Rep.

[67] Seaborne RA, Strauss J, Cocks M, Shepherd S, O’brien TD, van Someren KA (2018). Methylome of human skeletal muscle after acute & chronic resistance exercise training, detraining & retraining. Sci Data.

[68] Cheng S, Wang X, Zhang Q, He Y, Zhang X, Yang L (2020). Comparative Transcriptome Analysis Identifying the Different Molecular Genetic Markers Related to Production Performance and Meat Quality in Longissimus Dorsi Tissues of MG × STH and STH Sheep. Genes (Basel).

[69] Lei C, Du F, Sun L, Li T, Li T, Min Y (2017). MIR-143 & MIR-145 inhibit gastric cancer cell migration & metastasis by suppressing MYO6. Cell Death Dis.

[70] He H, Bronisz A, Liyanarachchi S, Nagy R, Li W, Huang Y (2013). *SRGAP1* is a candidate gene for papillary thyroid carcinoma susceptibility. J Clin Endocrinol Metab.

[71] Melchionda S, Ahituv N, Bisceglia L, Sobe T, Glaser F, Rabionet R (2001). MYO6, the human homologue of the gene responsible for deafness in Snell’s waltzer mice, is mutated in autosomal dominant nonsyndromic hearing loss. Am J Hum Genet.

[72] Rodriguez-Murillo L, Xu B, Roos JL, Abecasis GR, Gogos JA, Karayiorgou M (2014). Fine mapping on chromosome 13q32-34 and brain expression analysis implicates MYO16 in schizophrenia. Neuropsychopharmacology..

[73] Shia J, Zhang L, Shike M, Guo M, Stadler Z, Xiong X (2013). Secondary mutation in a coding mononucleotide tract in MSH6 causes loss of immunoexpression of MSH6 in colorectal carcinomas with MLH1/PMS2 deficiency. Mod Pathol.

[74] Hendriks YMC, Wagner A, Morreau H, Menko F, Stormorken A, Quehenberger F (2004). Cancer risk in hereditary nonpolyposis colorectal cancer due to MSH6 mutations: impact on counseling and surveillance. Gastroenterology..

[75] Bugyi B, Kengyel A. Myosin XVI: Advances in Experimental Medicine and Biology. Springer; 2020. p. 405–19. 10.1007/978-3-030-38062-5_18. 10.1007/978-3-030-38062-5_18 32451869

[76] Yoshida GM, Barria A, Cáceres G, Correa K, Jedlicki A, Cadiz MI (2019). Genome-wide patterns of population structure and linkage disequilibrium in farmed Nile tilapia (Oreochromis niloticus). Front Genet.

[77] Sargolzaei M, Chesnais JP, Schenkel FS (2014). A new approach for efficient genotype imputation using information from relatives. BMC Genomics.

[78] Yang J, Lee SH, Goddard ME, Visscher PM (2011). GCTA: a tool for genome-wide complex trait analysis. Am J Hum Genet.

[79] Yang J, Benyamin B, McEvoy BP, Gordon S, Henders AK, Nyholt DR (2010). Common SNPs explain a large proportion of the heritability for human height. Nat Genet.

