# Supplementary figures and images for: Multi-trait GWAS using imputed high-density genotypes from whole-genome sequencing identifies genes associated with body traits in Nile tilapia
Source: BMC Genomics. 2021 Jan 15;22:57. doi: 10.1186/s12864-020-07341-z (PMC7811220; doi:10.1186/s12864-020-07341-z)

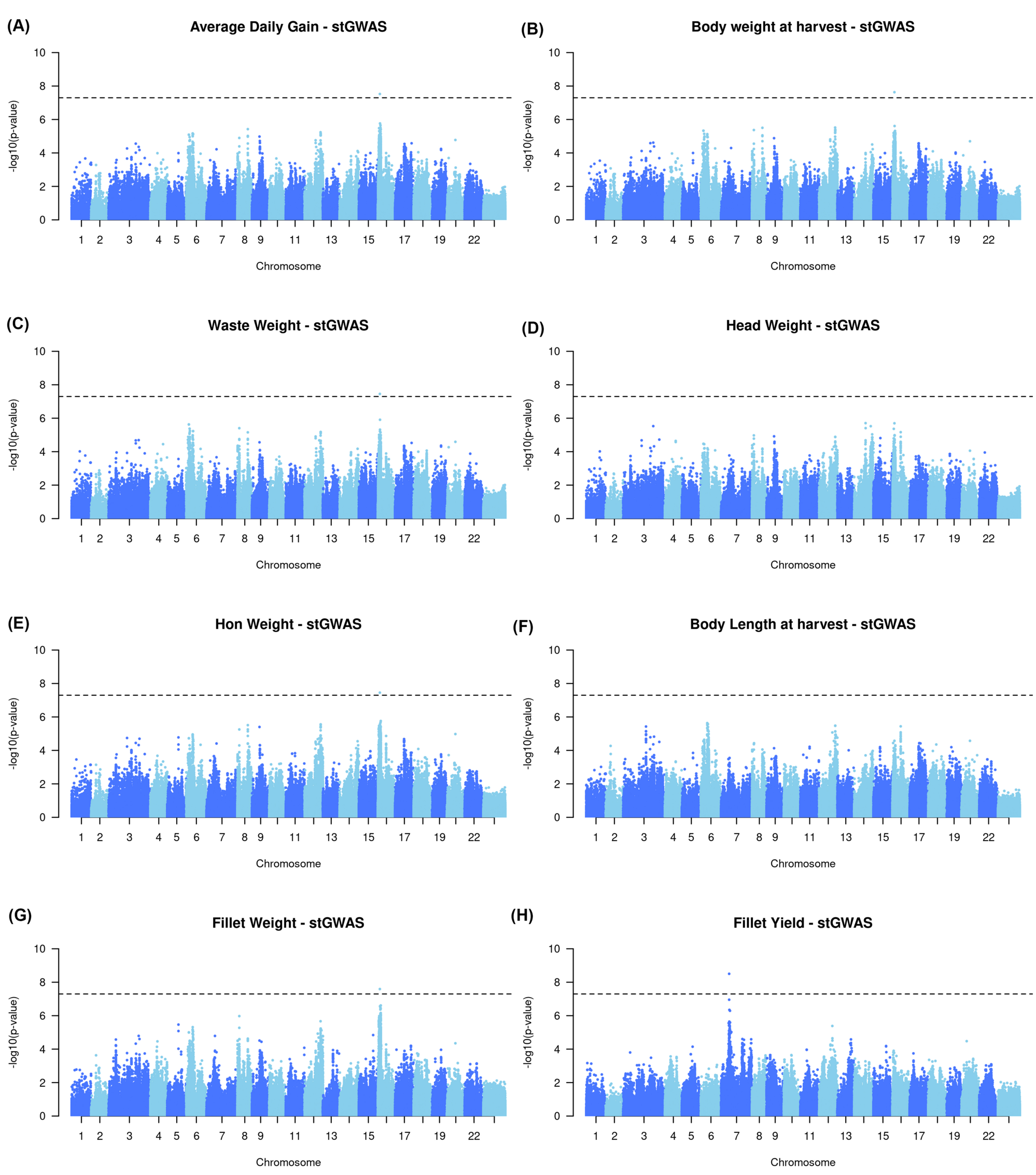

Supplement: Supplementary file 1 — Additional file 1: Supplementary Fig. 1. Manhattan plot for single-trait GWAS (stGWAS) for body traits in Nile tilapia. Manhattan plots of SNPs associated with: (A) Average daily gain. (B) Body weight at harvest. (C) Waste weight. (D) Head weight. (E) Gutted head-on weight. (F) Body length at harvest. (G) Fillet weight. (H) Fillet yield. The x-axis presents genomic coordinates along chromosomes 1–23 in Nile tilapia. On the y-axis the negative logarithm of the SNPs associated p-value is displayed. The dashed black line represents the genome-wide significance threshold after Bonferroni correction (−log10 (p-value > 7.30e-8)). [file 12864_2020_7341_MOESM1_ESM.pdf]
