# Supplementary material for: Multi-trait GWAS using imputed high-density genotypes from whole-genome sequencing identifies genes associated with body traits in Nile tilapia
Source: BMC Genomics. 2021 Jan 15;22:57. doi: 10.1186/s12864-020-07341-z (PMC7811220; doi:10.1186/s12864-020-07341-z)
Supplement: Supplementary file 2 — Additional file 2: Supplementary Table 1. Summary results from genotype quality control of whole-genome sequence (WGS), imputed WGS genotypes, and 50 K single nucleotide polymorphism (SNP) chip for Nile tilapia. [file 12864_2020_7341_MOESM2_ESM.docx]

Additional file 2: **Supplementary Table 1.** Summary results from genotype quality control of whole-genome sequence (WGS), imputed WGS, and 50K single nucleotide polymorphism (SNP) chips for Nile tilapia.

| Parameters | Genotypes data sets | | |
| --- | --- | --- | --- |
|  | **WGS^1^** | **Imputed WGS^2^** | **50K^3^** |
| N samples | 143 | 1,309 | 1,309 |
| Initial SNPs | 26,650,009 | 1,324,420 | 43,271 |
| Minor allele frequency | 7,788,652 | 253,076 | 4,114 |
| Call-rate | 12,061,149 | - | 3,665 |
| Hardy-Weinberg equilibrium | 1,789,157 | 78,850 | 5,905 |
| Final SNPs | 5,011,051 | 992,494 | 29,587 |

^1^Minor allele frequency (MAF) < 0.01, call-rate < 0.80 and Hardy-Weinberg equilibrium (HWE) < 1e-^8^; ^2^MAF < 0.05 and HWE < 1e-^8^; ^3^MAF < 0.01, call-rate <0.80 and HWE < 1e-^6^;
