# Supplementary material for: Multi-trait GWAS using imputed high-density genotypes from whole-genome sequencing identifies genes associated with body traits in Nile tilapia
Source: BMC Genomics. 2021 Jan 15;22:57. doi: 10.1186/s12864-020-07341-z (PMC7811220; doi:10.1186/s12864-020-07341-z)
Supplement: Supplementary file 3 — Additional file 3: Supplementary Table 2. Genomic regions and candidate genes for all lead SNPs associated with body traits based on multi-trait GWAS for Nile tilapia [file 12864_2020_7341_MOESM3_ESM.docx]

Additional file 3: **Supplementary Table 2.** Genomic regions and candidate genes for all lead SNP associated with body traits based on multi-trait GWAS for Nile tilapia.

| **Marker** | **LG^1^** | **Position^2^** | **Allele** | **MAF^3^** | | **p-value** | **Genes^4^** |
| --- | --- | --- | --- | --- | --- | --- | --- |
| **Average daily gain** | | | | | | | |
| **1:39153024** | 1 | 39153024 | [G/A] | 0.052 | 2.82E-11 | | HDGFL3, CCDC102A |
| **1:39193509** | 1 | 39193509 | [A/G] | 0.052 | 2.82E-11 | | HDGFL3, CCDC102A, NADSYN1 |
| **1:39558113** | 1 | 39558113 | [G/A] | 0.052 | 2.82E-11 | | CCNE1, ZNF536 |
| **1:39628599** | 1 | 39628599 | [A/G] | 0.052 | 2.82E-11 | | ZNF536 |
| **3:47327036** | 3 | 47327036 | [A/G] | 0.113 | 6.77E-11 | | Uncharacterized |
| **4:24859012** | 4 | 24859012 | [C/A] | 0.059 | 3.38E-11 | | USP31, HS3ST4 |
| **5:6037462** | 5 | 6037462 | [T/G] | 0.055 | 6.15E-09 | | DDIT3, MARS, ARHGAP9, GLI1, R3HDM2 |
| **6:26052504** | 6 | 26052504 | [A/C] | 0.060 | 1.93E-08 | | Uncharacterized |
| **7:54783508** | 7 | 54783508 | [A/G] | 0.051 | 8.36E-09 | | GTF3C6, FAM107B |
| **8:396284** | 8 | 396284 | [T/C] | 0.108 | 3.92E-09 | | Uncharacterized |
| **9:16267509** | 9 | 16267509 | [C/T] | 0.077 | 1.68E-10 | | PLXDC2, MALRD1, WDR60, ESYT2 |
| **9:16435917** | 9 | 16435917 | [C/T] | 0.075 | 4.76E-09 | | WDR60, ESYT2, NCAPG2, PTPRN2 |
| **12:24557870** | 12 | 24557870 | [A/G] | 0.069 | 2.63E-15 | | HSD17B4, DMXL1, SEMA6A |
| **12:24557984** | 12 | 24557984 | [T/C] | 0.069 | 2.63E-15 | | HSD17B4, DMXL1, SEMA6A |
| **13:21626153** | 13 | 21626153 | [A/G] | 0.101 | 3.80E-08 | | GTH-RII, GTF2A1L, STON1, PPP1R21, FOXN2, FBXO11, MSH6, KCNK12, GOLGA4, ITGA9 |
| **13:21626426** | 13 | 21626426 | [T/C] | 0.101 | 3.80E-08 | | GTH-RII, GTF2A1L, STON1, PPP1R21, FOXN2, FBXO11, MSH6, KCNK12, GOLGA4, ITGA9 |
| **15:14410587** | 15 | 14410587 | [A/G] | 0.088 | 4.86E-08 | | GPHN, FUT8 |
| **15:14413544** | 15 | 14413544 | [A/C] | 0.088 | 4.86E-08 | | GPHN, FUT8 |
| **15:14413799** | 15 | 14413799 | [T/C] | 0.088 | 4.86E-08 | | GPHN, FUT8 |
| **15:14413803** | 15 | 14413803 | [T/G] | 0.088 | 4.86E-08 | | GPHN, FUT8 |
| **15:14414140** | 15 | 14414140 | [C/T] | 0.088 | 4.86E-08 | | GPHN, FUT8 |
| **15:14414368** | 15 | 14414368 | [T/G] | 0.088 | 4.86E-08 | | GPHN, FUT8 |
| **15:14419133** | 15 | 14419133 | [C/T] | 0.088 | 4.86E-08 | | GPHN, FUT8 |
| **15:14420897** | 15 | 14420897 | [C/T] | 0.088 | 4.86E-08 | | GPHN, FUT8 |
| **15:14421396** | 15 | 14421396 | [G/A] | 0.088 | 4.86E-08 | | GPHN, FUT8 |
| **15:14421648** | 15 | 14421648 | [C/A] | 0.088 | 4.86E-08 | | GPHN, FUT8 |
| **15:14421941** | 15 | 14421941 | [G/A] | 0.088 | 4.86E-08 | | GPHN, FUT8 |
| **15:14423479** | 15 | 14423479 | [A/G] | 0.088 | 4.86E-08 | | GPHN, FUT8 |
| **15:14457958** | 15 | 14457958 | [C/T] | 0.088 | 4.86E-08 | | GPHN, FUT8 |
| **15:14651060** | 15 | 14651060 | [G/A] | 0.090 | 1.15E-09 | | TMEM121, SLC24A4 |
| **15:14651117** | 15 | 14651117 | [C/A] | 0.090 | 1.15E-09 | | TMEM121, SLC24A4 |
| **15:14662118** | 15 | 14662118 | [A/C] | 0.090 | 1.15E-09 | | TMEM121, SLC24A4 |
| **16:20105934** | 16 | 20105934 | [G/A] | 0.053 | 1.07E-09 | | ABHD13, TNFSF13B, MYO16, IRS2, COL4A1 |
| **16:20116545** | 16 | 20116545 | [T/C] | 0.053 | 1.07E-09 | | ABHD13, TNFSF13B, MYO16, IRS2, COL4A1 |
| **17:20586593** | 17 | 20586593 | [G/T] | 0.101 | 3.49E-09 | | Uncharacterized |
| **17:20601790** | 17 | 20601790 | [T/C] | 0.101 | 3.49E-09 | | Uncharacterized |
| **18:27368147** | 18 | 27368147 | [A/G] | 0.098 | 1.60E-10 | | Uncharacterized |
| **19:11094375** | 19 | 11094375 | [T/C] | 0.054 | 7.95E-09 | | TMEM260, KTN1, MSH4, VCPKMT, ASPG, KIF26A |
| **19:20435177** | 19 | 20435177 | [T/C] | 0.065 | 2.84E-08 | | MYCT1, SYNE1, CCDC170, RMND1, TRNAL-UAA, SHPRH |
| **22:11635404** | 22 | 11635404 | [C/A] | 0.083 | 1.49E-08 | | PITHD1, ELOA, RPL11, EF-1A |
| **22:11998439** | 22 | 11998439 | [G/A] | 0.059 | 2.80E-12 | | CFAP69, KDM1B, DPY19L4, INTS8, GDF6, PKHD1L1, NUDCD1 |
| Body weight at harvest | | | | | | | |
| **1:39153024** | 1 | 39153024 | [G/A] | 0.052 | 2.44E-10 | | HDGFL3, CCDC102A |
| **1:39193509** | 1 | 39193509 | [A/G] | 0.052 | 2.44E-10 | | HDGFL3, CCDC102A, NADSYN1 |
| **1:39558113** | 1 | 39558113 | [G/A] | 0.052 | 2.44E-10 | | CCNE1, ZNF536 |
| **1:39628599** | 1 | 39628599 | [A/G] | 0.052 | 2.44E-10 | | ZNF536 |
| **3:47050238** | 3 | 47050238 | [G/A] | 0.111 | 2.55E-10 | | Uncharacterized |
| **3:47051526** | 3 | 47051526 | [C/T] | 0.111 | 2.55E-10 | | Uncharacterized |
| **3:47051658** | 3 | 47051658 | [C/T] | 0.111 | 2.55E-10 | | Uncharacterized |
| **3:47064569** | 3 | 47064569 | [A/G] | 0.111 | 2.55E-10 | | Uncharacterized |
| **3:47128152** | 3 | 47128152 | [T/C] | 0.111 | 2.55E-10 | | TLR2 |
| **3:47130072** | 3 | 47130072 | [A/G] | 0.111 | 2.55E-10 | | TLR2 |
| **3:47130123** | 3 | 47130123 | [G/T] | 0.111 | 2.55E-10 | | TLR2 |
| **3:47130132** | 3 | 47130132 | [C/T] | 0.111 | 2.55E-10 | | TLR2 |
| **3:47132237** | 3 | 47132237 | [T/C] | 0.111 | 2.55E-10 | | TLR2 |
| **3:47132276** | 3 | 47132276 | [T/C] | 0.111 | 2.55E-10 | | TLR2 |
| **3:47132284** | 3 | 47132284 | [C/T] | 0.111 | 2.55E-10 | | TLR2 |
| **3:47132357** | 3 | 47132357 | [C/A] | 0.111 | 2.55E-10 | | TLR2 |
| **3:47132400** | 3 | 47132400 | [G/A] | 0.111 | 2.55E-10 | | TLR2 |
| **3:47132429** | 3 | 47132429 | [A/G] | 0.111 | 2.55E-10 | | TLR2 |
| **3:47132474** | 3 | 47132474 | [A/G] | 0.111 | 2.55E-10 | | TLR2 |
| **3:47138842** | 3 | 47138842 | [T/C] | 0.111 | 2.55E-10 | | TLR2 |
| **3:47142840** | 3 | 47142840 | [A/G] | 0.111 | 2.55E-10 | | TLR2 |
| **4:35484394** | 4 | 35484394 | [T/C] | 0.097 | 5.60E-09 | | CDC27 |
| **5:6037462** | 5 | 6037462 | [T/G] | 0.055 | 1.10E-08 | | DDIT3, MARS, ARHGAP9, GLI1, R3HDM2 |
| **6:4119361** | 6 | 4119361 | [T/C] | 0.127 | 3.13E-08 | | ERO1B, TBCE, RBM34 |
| **7:54783508** | 7 | 54783508 | [A/G] | 0.051 | 3.58E-08 | | GTF3C6, FAM107B |
| **8:542536** | 8 | 542536 | [A/G] | 0.109 | 8.55E-10 | | Uncharacterized |
| **8:542852** | 8 | 542852 | [A/G] | 0.109 | 8.55E-10 | | Uncharacterized |
| **8:543350** | 8 | 543350 | [G/A] | 0.109 | 8.55E-10 | | Uncharacterized |
| **8:543673** | 8 | 543673 | [A/G] | 0.109 | 8.55E-10 | | Uncharacterized |
| **8:544553** | 8 | 544553 | [C/A] | 0.109 | 8.55E-10 | | Uncharacterized |
| **8:544584** | 8 | 544584 | [T/C] | 0.109 | 8.55E-10 | | Uncharacterized |
| **8:544594** | 8 | 544594 | [G/T] | 0.109 | 8.55E-10 | | Uncharacterized |
| **8:545011** | 8 | 545011 | [T/C] | 0.109 | 8.55E-10 | | Uncharacterized |
| **8:545337** | 8 | 545337 | [A/G] | 0.109 | 8.55E-10 | | Uncharacterized |
| **8:545642** | 8 | 545642 | [C/T] | 0.109 | 8.55E-10 | | Uncharacterized |
| **8:545833** | 8 | 545833 | [T/C] | 0.109 | 8.55E-10 | | Uncharacterized |
| **8:546104** | 8 | 546104 | [C/A] | 0.109 | 8.55E-10 | | Uncharacterized |
| **8:546281** | 8 | 546281 | [G/A] | 0.109 | 8.55E-10 | | Uncharacterized |
| **8:546414** | 8 | 546414 | [G/A] | 0.109 | 8.55E-10 | | Uncharacterized |
| **8:546879** | 8 | 546879 | [A/G] | 0.109 | 8.55E-10 | | Uncharacterized |
| **8:546888** | 8 | 546888 | [G/T] | 0.109 | 8.55E-10 | | Uncharacterized |
| **8:549599** | 8 | 549599 | [T/C] | 0.109 | 8.55E-10 | | Uncharacterized |
| **8:560038** | 8 | 560038 | [C/T] | 0.109 | 8.55E-10 | | Uncharacterized |
| **8:561805** | 8 | 561805 | [C/T] | 0.109 | 8.55E-10 | | Uncharacterized |
| **8:567773** | 8 | 567773 | [C/T] | 0.109 | 8.55E-10 | | Uncharacterized |
| **8:567891** | 8 | 567891 | [C/T] | 0.109 | 8.55E-10 | | Uncharacterized |
| **9:16267509** | 9 | 16267509 | [C/T] | 0.077 | 3.71E-09 | | PLXDC2, MALRD1, WDR60, ESYT2 |
| **12:24557870** | 12 | 24557870 | [A/G] | 0.069 | 4.06E-15 | | HSD17B4, DMXL1, SEMA6A |
| **12:24557984** | 12 | 24557984 | [T/C] | 0.069 | 4.06E-15 | | HSD17B4, DMXL1, SEMA6A |
| **15:14651060** | 15 | 14651060 | [G/A] | 0.090 | 9.84E-09 | | TMEM121, SLC24A4 |
| **15:14651117** | 15 | 14651117 | [C/A] | 0.090 | 9.84E-09 | | TMEM121, SLC24A4 |
| **15:14662118** | 15 | 14662118 | [A/C] | 0.090 | 9.84E-09 | | TMEM121, SLC24A4 |
| **16:20105934** | 16 | 20105934 | [G/A] | 0.053 | 1.99E-09 | | ABHD13, TNFSF13B, MYO16, IRS2, COL4A1 |
| **16:20116545** | 16 | 20116545 | [T/C] | 0.053 | 1.99E-09 | | ABHD13, TNFSF13B, MYO16, IRS2, COL4A1 |
| **17:19609147** | 17 | 19609147 | [C/T] | 0.102 | 4.10E-08 | | LG17H12ORF66, TMEM19, RAB3IP, PROSER2, UPF2, SLC35E3, NUP107, MDM2 |
| **17:19612729** | 17 | 19612729 | [T/C] | 0.102 | 4.10E-08 | | LG17H12ORF66, TMEM19, RAB3IP, PROSER2, UPF2, SLC35E3, NUP107, MDM2 |
| **18:27368147** | 18 | 27368147 | [A/G] | 0.098 | 3.82E-10 | | Uncharacterized |
| **19:11468492** | 19 | 11468492 | [C/T] | 0.053 | 1.42E-09 | | TMEM179, UBR1, SERPINA10, DDX24, OTUB2, CCDC197, ASB2, FAM181A, PRIMA1UNC79, BTBD7, ITPK1 |
| **22:11635404** | 22 | 11635404 | [C/A] | 0.083 | 6.01E-09 | | PITHD1, ELOA, RPL11, EF-1A |
| **Head weight** | | | | | | | |
| **1:39153024** | 1 | 39153024 | [G/A] | 0.052 | 3.47E-10 | | HDGFL3, CCDC102A |
| **1:39193509** | 1 | 39193509 | [A/G] | 0.052 | 3.47E-10 | | HDGFL3, CCDC102A, NADSYN1 |
| **1:39558113** | 1 | 39558113 | [G/A] | 0.052 | 3.47E-10 | | CCNE1, ZNF536 |
| **1:39628599** | 1 | 39628599 | [A/G] | 0.052 | 3.47E-10 | | ZNF536 |
| **3:50439330** | 3 | 50439330 | [C/T] | 0.109 | 3.11E-13 | | Uncharacterized |
| **3:50439365** | 3 | 50439365 | [T/C] | 0.109 | 3.11E-13 | | Uncharacterized |
| **4:17899270** | 4 | 17899270 | [G/T] | 0.051 | 2.45E-10 | | Uncharacterized |
| **4:17917146** | 4 | 17917146 | [T/G] | 0.051 | 2.45E-10 | | Uncharacterized |
| **5:6040474** | 5 | 6040474 | [T/C] | 0.059 | 3.04E-10 | | MARS, ARHGAP9, GLI1, R3HDM2 |
| **5:6040483** | 5 | 6040483 | [C/T] | 0.059 | 3.04E-10 | | MARS, ARHGAP9, GLI1, R3HDM2 |
| **5:6042034** | 5 | 6042034 | [A/G] | 0.059 | 3.04E-10 | | MARS, ARHGAP9, GLI1, R3HDM2 |
| **7:60999336** | 7 | 60999336 | [A/G] | 0.065 | 4.61E-09 | | URI1, RXYLT1, SRGAP1, CCDC113, PARP12, TRNAG-UCC |
| **7:61003333** | 7 | 61003333 | [G/T] | 0.065 | 4.61E-09 | | URI1, RXYLT1, SRGAP1, CCDC113, PARP12, TRNAG-UCC |
| **7:61005198** | 7 | 61005198 | [T/C] | 0.065 | 4.61E-09 | | URI1, RXYLT1, SRGAP1, CCDC113, PARP12, TRNAG-UCC |
| **8:396284** | 8 | 396284 | [T/C] | 0.108 | 3.61E-09 | | Uncharacterized |
| **9:16433765** | 9 | 16433765 | [T/C] | 0.074 | 4.09E-09 | | WDR60, ESYT2, NCAPG2, PTPRN2 |
| **10:32346497** | 10 | 32346497 | [C/T] | 0.056 | 1.50E-08 | | Uncharacterized |
| **10:32346502** | 10 | 32346502 | [A/G] | 0.056 | 1.50E-08 | | Uncharacterized |
| **11:19628608** | 11 | 19628608 | [T/G] | 0.069 | 2.37E-08 | | INO80C, GALNT1 |
| **12:24557870** | 12 | 24557870 | [A/G] | 0.069 | 4.08E-15 | | HSD17B4, DMXL1, SEMA6A |
| **12:24557984** | 12 | 24557984 | [T/C] | 0.069 | 4.08E-15 | | HSD17B4, DMXL1, SEMA6A |
| **13:29981822** | 13 | 29981822 | [A/G] | 0.056 | 1.50E-08 | | Uncharacterized |
| **14:64504** | 14 | 64504 | [T/C] | 0.056 | 1.50E-08 | | USF1 |
| **14:64506** | 14 | 64506 | [T/C] | 0.056 | 1.50E-08 | | USF1 |
| **14:64730** | 14 | 64730 | [G/A] | 0.056 | 1.50E-08 | | USF1 |
| **15:38529741** | 15 | 38529741 | [T/G] | 0.056 | 1.50E-08 | | CARD16 |
| **16:17284162** | 16 | 17284162 | [G/A] | 0.057 | 1.67E-08 | | ADAM23, GPR1, NDUFS1, INO80D, DOCK9 |
| **18:27360037** | 18 | 27360037 | [C/A] | 0.114 | 1.61E-08 | | Uncharacterized |
| **19:20435177** | 19 | 20435177 | [T/C] | 0.065 | 4.77E-08 | | MYCT1, SYNE1, CCDC170, RMND1, TRNAL-UAA, SHPRH |
| **20:18209251** | 20 | 18209251 | [G/A] | 0.056 | 7.30E-09 | | TK, LRRC23, UBA1 |
| **22:11998439** | 22 | 11998439 | [G/A] | 0.059 | 7.34E-10 | | CFAP69, KDM1B, DPY19L4, INTS8, GDF6, PKHD1L1, NUDCD1 |
| **Body length at harvest** | | | | | | | |
| **1:39153024** | 1 | 39153024 | [G/A] | 0.052 | 3.15E-12 | | HDGFL3, CCDC102A |
| **1:39193509** | 1 | 39193509 | [A/G] | 0.052 | 3.15E-12 | | HDGFL3, CCDC102A, NADSYN1 |
| **1:39558113** | 1 | 39558113 | [G/A] | 0.052 | 3.15E-12 | | CCNE1, ZNF536 |
| **1:39628599** | 1 | 39628599 | [A/G] | 0.052 | 3.15E-12 | | ZNF536 |
| **3:47137003** | 3 | 47137003 | [A/C] | 0.110 | 6.91E-12 | | TLR2 |
| **4:17982574** | 4 | 17982574 | [A/G] | 0.066 | 2.38E-10 | | SREBF1, TOM1L2, DRC3 |
| **5:6044430** | 5 | 6044430 | [T/C] | 0.069 | 2.18E-09 | | MARS, ARHGAP9, GLI1, R3HDM2 |
| **6:32690869** | 6 | 32690869 | [C/T] | 0.101 | 2.61E-09 | | ASPSCR1, NOTUM, TMC6, TMC8, TK1 |
| **7:54783508** | 7 | 54783508 | [A/G] | 0.051 | 9.64E-09 | | GTF3C6, FAM107B |
| **8:542536** | 8 | 542536 | [A/G] | 0.109 | 4.10E-08 | | Uncharacterized |
| **8:542852** | 8 | 542852 | [A/G] | 0.109 | 4.10E-08 | | Uncharacterized |
| **8:543350** | 8 | 543350 | [G/A] | 0.109 | 4.10E-08 | | Uncharacterized |
| **8:543673** | 8 | 543673 | [A/G] | 0.109 | 4.10E-08 | | Uncharacterized |
| **8:544553** | 8 | 544553 | [C/A] | 0.109 | 4.10E-08 | | Uncharacterized |
| **8:544584** | 8 | 544584 | [T/C] | 0.109 | 4.10E-08 | | Uncharacterized |
| **8:544594** | 8 | 544594 | [G/T] | 0.109 | 4.10E-08 | | Uncharacterized |
| **8:545011** | 8 | 545011 | [T/C] | 0.109 | 4.10E-08 | | Uncharacterized |
| **8:545337** | 8 | 545337 | [A/G] | 0.109 | 4.10E-08 | | Uncharacterized |
| **8:545642** | 8 | 545642 | [C/T] | 0.109 | 4.10E-08 | | Uncharacterized |
| **8:545833** | 8 | 545833 | [T/C] | 0.109 | 4.10E-08 | | Uncharacterized |
| **8:546104** | 8 | 546104 | [C/A] | 0.109 | 4.10E-08 | | Uncharacterized |
| **8:546281** | 8 | 546281 | [G/A] | 0.109 | 4.10E-08 | | Uncharacterized |
| **8:546414** | 8 | 546414 | [G/A] | 0.109 | 4.10E-08 | | Uncharacterized |
| **8:546879** | 8 | 546879 | [A/G] | 0.109 | 4.10E-08 | | Uncharacterized |
| **8:546888** | 8 | 546888 | [G/T] | 0.109 | 4.10E-08 | | Uncharacterized |
| **8:549599** | 8 | 549599 | [T/C] | 0.109 | 4.10E-08 | | Uncharacterized |
| **8:560038** | 8 | 560038 | [C/T] | 0.109 | 4.10E-08 | | Uncharacterized |
| **8:561805** | 8 | 561805 | [C/T] | 0.109 | 4.10E-08 | | Uncharacterized |
| **8:567773** | 8 | 567773 | [C/T] | 0.109 | 4.10E-08 | | Uncharacterized |
| **8:567891** | 8 | 567891 | [C/T] | 0.109 | 4.10E-08 | | Uncharacterized |
| **9:27601979** | 9 | 27601979 | [A/G] | 0.054 | 4.28E-08 | | Uncharacterized |
| **11:19476671** | 11 | 19476671 | [T/G] | 0.063 | 1.60E-09 | | TBRG4, ATP4A, TRNAR-ACG |
| **12:27146675** | 12 | 27146675 | [C/T] | 0.079 | 1.96E-13 | | GPX8, MCIDAS, ISCA1, PSAT1 |
| **13:23915471** | 13 | 23915471 | [A/G] | 0.050 | 3.07E-08 | | EGLN1, TSNAX, DISC1, SIPA1L2 |
| **14:18442958** | 14 | 18442958 | [A/G] | 0.059 | 3.08E-08 | | FCHSD2, YIF1B, BLOC1S3 |
| **15:13929129** | 15 | 13929129 | [T/C] | 0.082 | 5.00E-08 | | KCNK3, SLC35F6, HLX |
| **16:20105934** | 16 | 20105934 | [G/A] | 0.053 | 3.34E-11 | | ABHD13, TNFSF13B, MYO16, IRS2, COL4A1 |
| **17:25198694** | 17 | 25198694 | [G/A] | 0.098 | 1.28E-09 | | POMGNT1 |
| **18:18194228** | 18 | 18194228 | [A/G] | 0.051 | 1.23E-09 | | EFCAB14, ZNF830 |
| **19:9747813** | 19 | 9747813 | [T/C] | 0.079 | 6.81E-09 | | EXD1, RASGRP1, SYNDIG1L, VSX2, LIN52 |
| **20:32775965** | 20 | 32775965 | [A/G] | 0.108 | 5.07E-08 | | GATA5, RBBP8NL, HCK, TM9SF4, PLAGL2 |
| **22:11998439** | 22 | 11998439 | [G/A] | 0.059 | 7.13E-18 | | CFAP69, KDM1B, DPY19L4, INTS8, GDF6, PKHD1L1, NUDCD1 |
| **22:11998439** | 22 | 11998439 | [G/A] | 0.059 | 7.13E-18 | | CFAP69, KDM1B, DPY19L4, INTS8, GDF6, PKHD1L1, NUDCD1 |
| **Hon weight** | | | | | | | |
| **1:39153024** | 1 | 39153024 | [G/A] | 0.052 | 1.28E-10 | | HDGFL3, CCDC102A |
| **1:39193509** | 1 | 39193509 | [A/G] | 0.052 | 1.28E-10 | | HDGFL3, CCDC102A, NADSYN1 |
| **1:39558113** | 1 | 39558113 | [G/A] | 0.052 | 1.28E-10 | | CCNE1, ZNF536 |
| **1:39628599** | 1 | 39628599 | [A/G] | 0.052 | 1.28E-10 | | ZNF536 |
| **3:47137003** | 3 | 47137003 | [A/C] | 0.110 | 1.17E-10 | | TLR2 |
| **4:35484394** | 4 | 35484394 | [T/C] | 0.097 | 1.32E-08 | | CDC27 |
| **5:6040474** | 5 | 6040474 | [T/C] | 0.059 | 3.85E-08 | | MARS, ARHGAP9, GLI1, R3HDM2 |
| **5:6040483** | 5 | 6040483 | [C/T] | 0.059 | 3.85E-08 | | MARS, ARHGAP9, GLI1, R3HDM2 |
| **5:6042034** | 5 | 6042034 | [A/G] | 0.059 | 3.85E-08 | | MARS, ARHGAP9, GLI1, R3HDM2 |
| **8:390896** | 8 | 390896 | [T/C] | 0.102 | 5.45E-08 | | Uncharacterized |
| **8:391578** | 8 | 391578 | [G/A] | 0.102 | 5.45E-08 | | Uncharacterized |
| **8:393162** | 8 | 393162 | [G/T] | 0.102 | 5.45E-08 | | Uncharacterized |
| **8:393184** | 8 | 393184 | [A/G] | 0.102 | 5.45E-08 | | Uncharacterized |
| **8:393250** | 8 | 393250 | [C/A] | 0.102 | 5.45E-08 | | Uncharacterized |
| **8:397651** | 8 | 397651 | [T/C] | 0.102 | 5.45E-08 | | Uncharacterized |
| **8:398534** | 8 | 398534 | [G/A] | 0.102 | 5.45E-08 | | Uncharacterized |
| **8:399361** | 8 | 399361 | [T/G] | 0.102 | 5.45E-08 | | Uncharacterized |
| **8:399394** | 8 | 399394 | [C/A] | 0.102 | 5.45E-08 | | Uncharacterized |
| **8:400216** | 8 | 400216 | [C/A] | 0.102 | 5.45E-08 | | Uncharacterized |
| **9:16328834** | 9 | 16328834 | [A/G] | 0.077 | 5.32E-08 | | PLXDC2, MALRD1, WDR60, ESYT2, NCAPG2, PTPRN2 |
| **12:24557870** | 12 | 24557870 | [A/G] | 0.069 | 9.89E-14 | | HSD17B4, DMXL1, SEMA6A |
| **12:24557984** | 12 | 24557984 | [T/C] | 0.069 | 9.89E-14 | | HSD17B4, DMXL1, SEMA6A |
| **15:23976527** | 15 | 23976527 | [T/C] | 0.090 | 5.18E-08 | | FILIP1, SENP6, MYO6 |
| **16:20105934** | 16 | 20105934 | [G/A] | 0.053 | 1.91E-10 | | ABHD13, TNFSF13B, MYO16, IRS2, COL4A1 |
| **17:19609147** | 17 | 19609147 | [C/T] | 0.102 | 3.82E-08 | | LG17H12ORF66, TMEM19, RAB3IP, PROSER2, UPF2, SLC35E3, NUP107, MDM2 |
| **17:19612729** | 17 | 19612729 | [T/C] | 0.102 | 3.82E-08 | | LG17H12ORF66, TMEM19, RAB3IP, PROSER2, UPF2, SLC35E3, NUP107, MDM2 |
| **18:27357619** | 18 | 27357619 | [T/C] | 0.115 | 3.56E-08 | | Uncharacterized |
| **19:11468492** | 19 | 11468492 | [C/T] | 0.053 | 4.89E-08 | | TMEM179, UBR1, SERPINA10, DDX24, OTUB2, CCDC197, ASB2, FAM181A, PRIMA1UNC79, BTBD7, ITPK1 |
| **22:11998439** | 22 | 11998439 | [G/A] | 0.059 | 5.59E-10 | | CFAP69, KDM1B, DPY19L4, INTS8, GDF6, PKHD1L1, NUDCD1 |
| **Waste weight** | | | | | | | |
| **1:39153024** | 1 | 39153024 | [G/A] | 0.052 | 8.43E-11 | | HDGFL3, CCDC102A |
| **1:39193509** | 1 | 39193509 | [A/G] | 0.052 | 8.43E-11 | | HDGFL3, CCDC102A, NADSYN1 |
| **1:39558113** | 1 | 39558113 | [G/A] | 0.052 | 8.43E-11 | | CCNE1, ZNF536 |
| **1:39628599** | 1 | 39628599 | [A/G] | 0.052 | 8.43E-11 | | ZNF536 |
| **3:47327036** | 3 | 47327036 | [A/G] | 0.113 | 1.63E-10 | | Uncharacterized |
| **4:34954060** | 4 | 34954060 | [T/C] | 0.084 | 5.49E-10 | | PSMD3, SAMD14 |
| **5:6040474** | 5 | 6040474 | [T/C] | 0.059 | 9.93E-09 | | MARS, ARHGAP9, GLI1, R3HDM2 |
| **5:6040483** | 5 | 6040483 | [C/T] | 0.059 | 9.93E-09 | | MARS, ARHGAP9, GLI1, R3HDM2 |
| **5:6042034** | 5 | 6042034 | [A/G] | 0.059 | 9.93E-09 | | MARS, ARHGAP9, GLI1, R3HDM2 |
| **6:4119361** | 6 | 4119361 | [T/C] | 0.127 | 1.63E-08 | | ERO1B, TBCE, RBM34 |
| **7:3766803** | 7 | 3766803 | [A/G] | 0.056 | 3.58E-08 | | Uncharacterized |
| **7:49374205** | 7 | 49374205 | [G/T] | 0.056 | 3.58E-08 | | RELN, TP53I11, CD82 |
| **7:49374211** | 7 | 49374211 | [G/A] | 0.056 | 3.58E-08 | | RELN, TP53I11, CD82 |
| **7:49374217** | 7 | 49374217 | [A/G] | 0.056 | 3.58E-08 | | RELN, TP53I11, CD82 |
| **7:49374223** | 7 | 49374223 | [T/G] | 0.056 | 3.58E-08 | | RELN, TP53I11, CD82 |
| **7:49374230** | 7 | 49374230 | [A/G] | 0.056 | 3.58E-08 | | RELN, TP53I11, CD82 |
| **7:49374233** | 7 | 49374233 | [G/T] | 0.056 | 3.58E-08 | | RELN, TP53I11, CD82 |
| **7:49374263** | 7 | 49374263 | [T/C] | 0.056 | 3.58E-08 | | RELN, TP53I11, CD82 |
| **7:49374277** | 7 | 49374277 | [A/G] | 0.056 | 3.58E-08 | | RELN, TP53I11, CD82 |
| **7:49374283** | 7 | 49374283 | [G/A] | 0.056 | 3.58E-08 | | RELN, TP53I11, CD82 |
| **8:3796164** | 8 | 3796164 | [G/A] | 0.113 | 5.08E-08 | | KCNJ2 |
| **9:14670077** | 9 | 14670077 | [C/T] | 0.059 | 5.22E-08 | | SLC4A2, ABCF2, CHPF2, SMARCD3 |
| **9:14674835** | 9 | 14674835 | [A/G] | 0.059 | 5.22E-08 | | SLC4A2, ABCF2, CHPF2, SMARCD3 |
| **10:32346497** | 10 | 32346497 | [C/T] | 0.056 | 3.58E-08 | | Uncharacterized |
| **10:32346502** | 10 | 32346502 | [A/G] | 0.056 | 3.58E-08 | | Uncharacterized |
| **11:19628608** | 11 | 19628608 | [T/G] | 0.069 | 3.87E-09 | | INO80C, GALNT1 |
| **12:24557870** | 12 | 24557870 | [A/G] | 0.069 | 2.51E-15 | | HSD17B4, DMXL1, SEMA6A |
| **12:24557984** | 12 | 24557984 | [T/C] | 0.069 | 2.51E-15 | | HSD17B4, DMXL1, SEMA6A |
| **13:29981822** | 13 | 29981822 | [A/G] | 0.056 | 3.58E-08 | | Uncharacterized |
| **14:64504** | 14 | 64504 | [T/C] | 0.056 | 3.58E-08 | | USF1 |
| **14:64506** | 14 | 64506 | [T/C] | 0.056 | 3.58E-08 | | USF1 |
| **14:64730** | 14 | 64730 | [G/A] | 0.056 | 3.58E-08 | | USF1 |
| **14:24825295** | 14 | 24825295 | [C/T] | 0.056 | 3.58E-08 | | Uncharacterized |
| **15:14651060** | 15 | 14651060 | [G/A] | 0.090 | 9.42E-10 | | TMEM121, SLC24A4 |
| **15:14651117** | 15 | 14651117 | [C/A] | 0.090 | 9.42E-10 | | TMEM121, SLC24A4 |
| **15:14662118** | 15 | 14662118 | [A/C] | 0.090 | 9.42E-10 | | TMEM121, SLC24A4 |
| **16:20105934** | 16 | 20105934 | [G/A] | 0.053 | 5.48E-11 | | ABHD13, TNFSF13B, MYO16, IRS2, COL4A1 |
| **16:20116545** | 16 | 20116545 | [T/C] | 0.053 | 5.48E-11 | | ABHD13, TNFSF13B, MYO16, IRS2, COL4A1 |
| **17:31075239** | 17 | 31075239 | [A/G] | 0.067 | 1.76E-08 | | ZMAT3, STXBP3, HENMT1, FAM102B, TM4SF4, |
| **18:17100458** | 18 | 17100458 | [T/C] | 0.056 | 1.67E-08 | | ZNF521, SS18, PSMA8, KCTD1 |
| **22:11998439** | 22 | 11998439 | [G/A] | 0.059 | 2.13E-12 | | CFAP69, KDM1B, DPY19L4, INTS8, GDF6, PKHD1L1, NUDCD1 |
| **Fillet waste** | | | | | | | |
| **3:15396071** | 3 | 15396071 | [A/G] | 0.092 | 1.17E-08 | | TRNAR-UCU, TRNAA-CGC, MSANTD1, HGFAC, DOK7 |
| **4:34954382** | 4 | 34954382 | [T/C] | 0.107 | 1.19E-14 | | PSMD3, SAMD14 |
| **4:34954397** | 4 | 34954397 | [A/G] | 0.107 | 1.19E-14 | | PSMD3, SAMD14 |
| **4:34958811** | 4 | 34958811 | [A/G] | 0.107 | 1.19E-14 | | PSMD3, SAMD14 |
| **4:34958990** | 4 | 34958990 | [G/A] | 0.107 | 1.19E-14 | | PSMD3, SAMD14 |
| **4:34959371** | 4 | 34959371 | [C/A] | 0.107 | 1.19E-14 | | PSMD3, SAMD14 |
| **4:34960461** | 4 | 34960461 | [T/C] | 0.107 | 1.19E-14 | | PSMD3, SAMD14 |
| **4:34963090** | 4 | 34963090 | [T/C] | 0.107 | 1.19E-14 | | PSMD3, SAMD14 |
| **5:29062243** | 5 | 29062243 | [G/A] | 0.058 | 3.45E-11 | | PRICKLE2, ADAMTS9, MAGI1 |
| **5:29062243** | 5 | 29062243 | [G/A] | 0.058 | 3.45E-11 | | PRICKLE2, ADAMTS9, MAGI1 |
| **6:6596302** | 6 | 6596302 | [A/G] | 0.065 | 3.97E-09 | | Uncharacterized |
| **8:396284** | 8 | 396284 | [T/C] | 0.108 | 8.77E-11 | | Uncharacterized |
| **12:24525556** | 12 | 24525556 | [A/G] | 0.062 | 2.38E-11 | | PRR16, HSD17B4, DMXL1, SEMA6A |
| **13:30002073** | 13 | 30002073 | [A/G] | 0.174 | 1.78E-18 | | Uncharacterized |
| **16:1683743** | 16 | 1683743 | [G/A] | 0.052 | 1.90E-08 | | KDELC1, TEX30, NEPRO, RALB |
| **18:27368147** | 18 | 27368147 | [A/G] | 0.098 | 2.11E-09 | | Uncharacterized |
| **22:11635404** | 22 | 11635404 | [C/A] | 0.083 | 1.15E-08 | | PITHD1, ELOA, RPL11, EF-1A |
| **Fillet yield** | | | | | | | |
| **6:33824877** | 6 | 33824877 | [T/G] | 0.055 | 4.50E-10 | | XYLT1, RPS15A, COQ7 |
| **12:26984411** | 12 | 26984411 | [G/A] | 0.066 | 2.39E-12 | | Uncharacterized |
| **13:17730096** | 13 | 17730096 | [C/A] | 0.113 | 2.01E-08 | | SLC25A16, ALOX5, ZFAND4, MARVELD1, AVPI1, MARCH8 |
| **13:17730605** | 13 | 17730605 | [C/A] | 0.113 | 2.01E-08 | | SLC25A16, ALOX5, ZFAND4, MARVELD1, AVPI1, MARCH8 |
| **14:30148797** | 14 | 30148797 | [G/T] | 0.056 | 3.01E-09 | | Uncharacterized |
| **16:12574352** | 16 | 12574352 | [G/A] | 0.051 | 3.84E-08 | | ZNF148, SLC12A8, HEG1, KANSL1L |

^1^Linkage group. ^2^In base pairs. ^3^Minor allele frequency. ^4^Genes found within a window of 100 kb downstream and upstream from the lead SNP using *Oreochromis niloticus* as the genome reference (O_niloticus_UMD_NMBU).
